# Hierarchical organization and molecular diffusion in gold nanorod/silica supercrystal nanocomposites†
†Electronic supplementary information (ESI) available. See DOI: 10.1039/c6nr00712k



**DOI:** 10.1039/c6nr00712k

**Published:** 2016-02-29

**Authors:** Cyrille Hamon, Marta N. Sanz-Ortiz, Evgeny Modin, Eric H. Hill, Leonardo Scarabelli, Andrey Chuvilin, Luis M. Liz-Marzán

**Affiliations:** a Bionanoplasmonics Laboratory , CIC biomaGUNE , Paseo de Miramón 182 , 20009 Donostia – San Sebastian , Spain . Email: llizmarzan@cicbiomagune.es; b Electron Microscopy and Image Processing Interdisciplinary Laboratory , Far Eastern Federal University , Sukhanova 8 , 690000 , Vladivostok , Russia; c Electron Microscopy Laboratory , CIC NanoGUNE Consolider , Tolosa Hiribidea , 76 , 20019 Donostia – San Sebastian , Spain; d Basque Foundation of Science , IKERBASQUE , 48013 Bilbao , Spain; e Biomedical Research Networking Center in Bioengineering , Biomaterials , and Nanomedicine , CIBER-BBN , Spain

## Abstract

Hierarchical organization of gold nanorods was previously obtained on a substrate, allowing precise control over the morphology of the assemblies and macroscale spatial arrangement. Herein, a thorough description of these gold nanorod assemblies and their orientation within supercrystals is presented together with a sol–gel technique to protect the supercrystals with mesoporous silica films. The internal organization of the nanorods in the supercrystals was characterized by combining focused ion beam ablation and scanning electron microscopy. A mesoporous silica layer is grown both over the supercrystals and between the individual lamellae of gold nanorods inside the structure. This not only prevented the detachment of the supercrystal from the substrate in water, but also allowed small molecule analytes to infiltrate the structure. These nanocomposite substrates show superior Raman enhancement in comparison with gold supercrystals without silica owing to improved accessibility of the plasmonic hot spots to analytes. The patterned supercrystal arrays with enhanced optical and mechanical properties obtained in this work show potential for the practical implementation of nanostructured devices in spatially resolved ultradetection of biomarkers and other analytes.

## Abstract 2

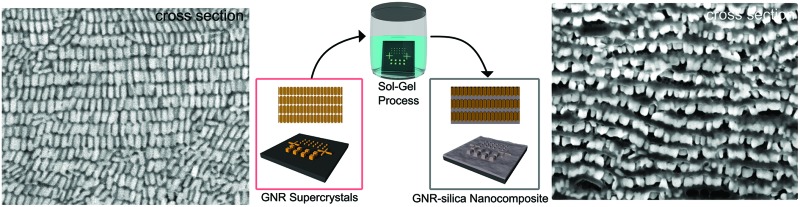
The internal organization of gold nanorod supercrystals is fully described as well as infiltration with silica and SERS performance.

## Introduction

The design of materials with hierarchical organization ranging from the nanoscale to the macroscale is of interest for the development of novel sensing platforms and optical devices.^1–5^ The organization of particles into self-assembled structures results in a material with increased overall size, dimensionality, and complexity, and the emergence of collective properties different from those of the individual components.^2,6^ Noble metal nanoparticles organized into supercrystalline structures (*i.e.* supercrystals) can lead to significant improvements in targeted plasmonic-sensing applications, as the amplification of optical signals characteristic of single metal nanoparticles is highly enhanced when collective plasmon coupling occurs.^7–9^

Despite the perceived advantages of hierarchically-organized metal nanoparticles, directing the macroscale organization of metal nanoparticles is still a major challenge.^10^ Nanoparticle self-assembly within a colloidal dispersion can be directed by specific (*e.g.* DNA base pairing) and non-specific interactions (*e.g.* electrostatic or capillary interactions), which occur at different length scales and should be accounted for when devising a hierarchically organized system.^11–13^ In the particular case of evaporative self-assembly, colloidal crystallization relies on a balance of nanoscale forces and their large scale deposition on the substrate is acted on by dragging flows.^14–19^ For example, when gold nanoparticles are deposited by drop casting on substrates, the colloidal particles are carried towards the pinned edge of the drying drop by the coffee ring effect.^20^ In this context, the process of gold nanoparticle self-assembly varies from the edge to the center of the drying suspension, resulting in the inhomogeneous distribution of the particles in terms of density, packing and organization.^21,22^ Although the drop-casting method is a convenient method to obtain gold supercrystals, the lack of reproducibility is a major limitation for its further use. To overcome the coffee ring effect, a variety of strategies have been proposed to control the multi-scale organization of metal nanoparticles, generally using serial lithographic techniques.^10,23,24^

An ideal fabrication method requires simple, cost-effective, and parallelizable processes.^2^ In this regard, with the ultimate aim to design a sensing platform, we recently demonstrated that patterned substrates can be fabricated by slow drying of gold nanoparticle colloids within micron-sized cavities from a polydimethysiloxane (PDMS) template.^25^ Although a variety of metal nanoparticles might be suitable building blocks, gold nanorods are one of the most relevant shapes for applications in biomedical technologies, plasmon-enhanced spectroscopy or optoelectronic devices, among others.^8,26–28^ They can be produced at a high yield with low size dispersity and their plasmonic properties can be precisely tuned through their size and aspect ratio and also by directed self-assembly. The organization of nanorods within the assemblies is highly relevant as it results in the formation of hotspots in the gap between nanoparticles that can enhance Raman scattering by adsorbed molecules.^29,30^ The suitability of these substrates for surface enhanced Raman scattering (SERS) detection was indeed demonstrated in a previous study.^25^ Hexagonal arrangement of the nanorods on the upper surface of the supercrystals as well as a superlattice of standing nanorods inside the structures were observed, but a detailed characterization study of the internal organization of the supercrystals is needed. As the structure of the material directs its functionality, knowledge of the internal organization of the supercrystal is highly relevant for designing more efficient plasmonic substrates. In the present work, we used the focused ion beam (FIB) technique in tandem with scanning electron microscopy (SEM) to characterize the internal organization of composite nanostructures.

Another key point to be considered for the use of these supercrystals as plasmonic substrates is the close packing of the nanoparticle building blocks and the resulting impermeability for analytes of interest, as recently reported.^31^ It has been shown that SERS enhancement by gold nanorod supercrystals is independent of the number of nanorod monolayers because the detection of the analyte takes place almost exclusively at the external surface of the supercrystals.^31^ Thus, new strategies are required that allow the diffusion of molecules in between the nanorods within the ordered structure so as to reach the hotspots and improve SERS detection. To address this issue, we show that infiltration of the supercrystal with mesoporous silica does improve analyte diffusion, resulting in substrates with improved performance as SERS substrates. We envision that detailed characterization of these composite substrates can have a rapid effect in improving SERS sensing applications, particularly for small-molecule analytes in complex liquids such as biofluids or plant and tissue extracts.

## Results and discussion

### Nanorod organization in confined volumes

**Fig. 1:**
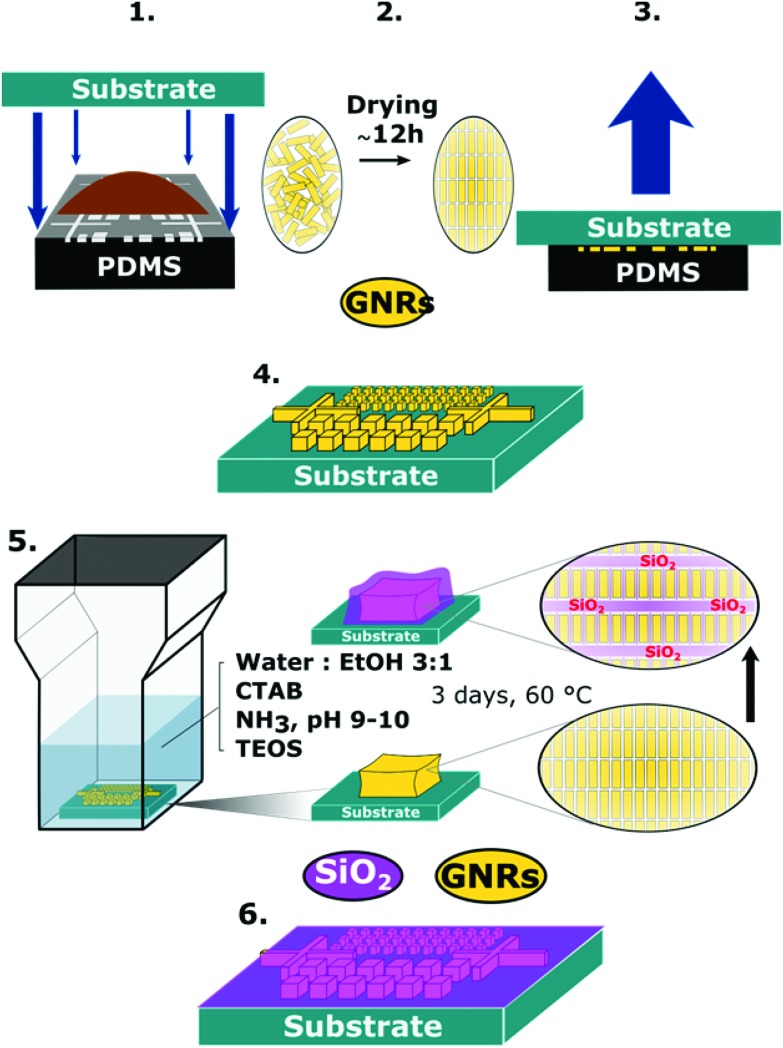
Schematic description of the preparation of the GNR-silica supercrystal nanocomposites. Upper panel: Preparation of the plasmonic substrate by the PDMS template-driven self-assembly process. (1) A 2 μL drop of GNR suspension was stamped with a PDMS template containing an array of micron-sized cavities. (2) The solvent was allowed to evaporate, leading to the assembly of GNRs into a smectic phase. (3) After drying, the template was removed. (4) Arrays of GNR supercrystals were obtained on the substrate. Lower panel: The process used to coat and infiltrate the GNR supercrystals with mesoporous silica. (5) The substrates were immersed in a growth solution containing TEOS, ammonia, and CTAB in 3 : 1 water : ethanol at 60 °C for 3 days. (6) Arrays of GNR supercrystals impregnated with mesoporous silica were obtained on the silicon substrate.

The organization of gold nanorods (GNRs) into supercrystals is dictated by external parameters that influence the drying time (*e.g.* temperature or humidity), the chemistry of the colloid (particle concentration and surface ligands), as well as the size and shape of the mold used to dry the particles.^10,16,32^ All GNRs used in this work had dimensions of 57 ± 5 nm in length and 17 ± 2 nm in width (ESI†) and were dried inside PDMS cavities having a fixed height of 4.8 μm and lateral dimensions of either 2, 6, 12 or 20 μm. Formation of GNR supercrystal arrays involved the functionalization of GNRs with (1-mercaptoundec-11-yl)hexa(ethyleneglycol) (MUDOL), which has been reported to induce GNR packing in a standing conformation.^25,33,34^ The GNR dispersion was dried, unless otherwise stated, under ambient conditions by confining the solution between a piece of silicon wafer and the PDMS template (Fig. 1a). Following supercrystal formation, the substrate was immersed in a solution containing silica precursors and surfactant micelles to create a mesoporous silica film that might infiltrate the supercrystal (Fig. 1b).

**Fig. 2:**
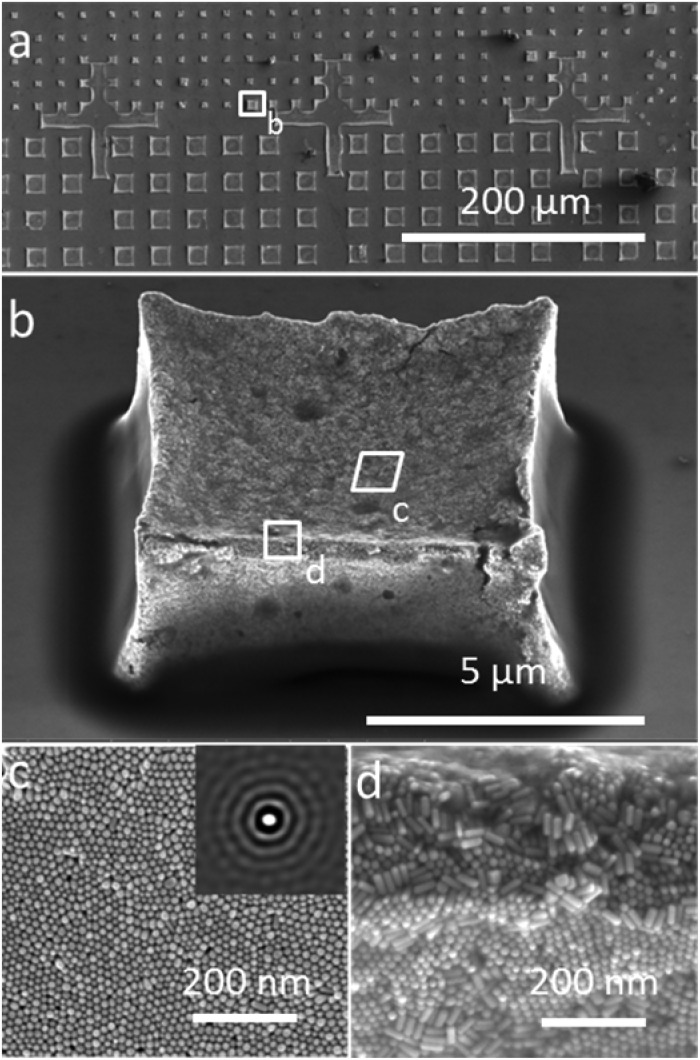
Hierarchically organized GNR supercrystals obtained by the PDMS driven self-assembly process. (a–d) SEM images taken at different magnifications of the supercrystal arrays deposited on Si (100). (c, d) The local hexagonal ordering of the nanorods was observed both on the top and at the edges of the supercrystals respectively. The inset in (c) shows autocorrelation analysis of the SEM image where up to five degrees of order can be distinguished.

**Fig. 3:**
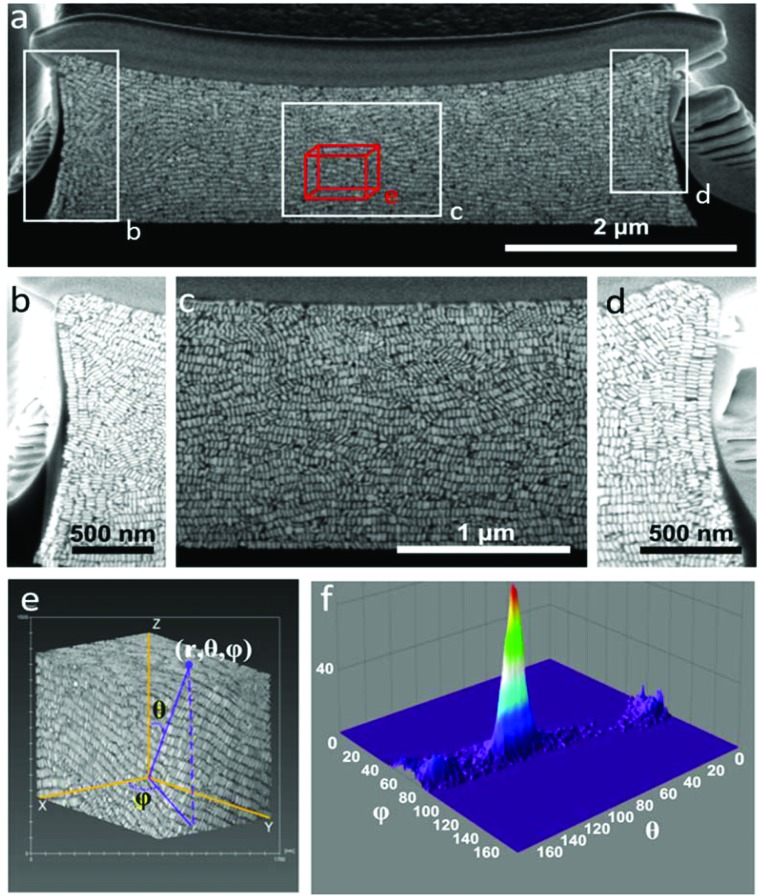
Organization of gold nanorods inside supercrystals after partial ablation by FIB. (a–d) SEM images at different magnifications. Images display GNR organization at the center and the edges of the supercrystal. (e) Three-dimensional reconstruction of a box within the supercrystal with a volume of 1.3 μm^3^, showing smectic organization. (f) Orientation scattering plot obtained from the analysis of the image in (e), revealing the distribution of orientation angles of the nanorods within the structure.

Our primary aim in this work was to achieve a detailed description of the GNR organization in these three dimensional structures. The supercrystals were initially imaged by SEM so that the hierarchical GNR organization could be determined at different magnifications and with high resolution, as shown in Fig. 2. Hexagonal GNR packing was observed, not only at the top of the layer but also on other facets, suggesting preferential orientation of the nanorods perpendicular to the outer surfaces of the supercrystals (Fig. 2). Local order was characterized by a 2D autocorrelation function (inset in Fig. 2c), which showed at least 5 maxima around the central spot, indicating that the local crystal order is preserved up to the 5^th^ neighbour on average. Further investigation of the internal structure was carried out by repetitive milling of a slice of a supercrystal by means of FIB, followed by high-resolution SEM (HRSEM) imaging of the supercrystal cross-section (Fig. S3†). This recursive procedure (Slice & View*™* method) allows 3D rendering of the internal structure (Fig. S4 and S5†). Fig. 3 shows a representative supercrystal cross section analyzed at different magnifications and a 3D volume reconstruction of the indicated region.

Whereas even inside the supercrystal most of the GNRs were found to be standing with their long axes pointing towards the silicon substrate, some GNR layers were found to orient perpendicular to the lateral facets (Fig. 3). Analysis of the GNR organization confirmed inter- and intra-lamellar long-range order, where multiple layers of GNRs retain a similar orientation toward the interface. Image analysis in three dimensions was also carried out in order to obtain a more quantitative measure of GNR organization, resulting in orientation scattering plots that revealed an average perpendicular orientation of the nanorods (92° ± 8°), with respect to the base of the substrate (Fig. 3f, Video 1†). The mechanism of GNR organization into supercrystals is still a matter of debate due to the challenge involved in the *in situ* investigation of the crystallization process. However, observation of the organization after drying gives valuable information on the parameters that have to be taken into account to obtain supercrystalline structures. It has been demonstrated that slow drying in a high humidity environment is crucial to reach a high level of organization.^11,18,28,35,36^ Additionally, in our previous work, CTAB-GNR and MUDOL-GNR were found to yield different supercrystal morphologies, suggesting the influence of surface chemistry on the self-assembly process,^31^ which can be attributed to the reduced electrostatic repulsion between nanorods in the case of MUDOL-GNR.^34,37^ The quasi-crystalline symmetry of MUDOL-GNR supercrystals has been attributed to the formation of ordered lamellae of packed nanorods in solution, which can then self-assemble in a second step on the substrate through a layer-by-layer process.^31,38^ We monitored the *in situ* formation of lamellae in the drying GNR colloid by dark field optical microscopy (Video 2†). Once a critical particle density was reached during solvent evaporation, large bright spots were observed, which we attribute to entropy-driven formation of ordered aggregates in solution.^14,39,40^ This observation would thus confirm a two-step process comprising formation of two-dimensional floating mono- or multi-layers of nanorods with parallel mutual arrangement, which can subsequently form supercrystals with increased dimensionality. In the case of the supercrystals displayed in Fig. 3, the use of a high concentration of GNRs hindered a direct observation of the assembly process by scattering techniques, but it is likely to occur *via* a similar two-step process. Thus, the observed organization at interfaces would result from monolayers forming at the air–water interface due to a locally higher evaporation rate.^32,41^ In this case, the evaporation was carried out over 12 h, but the effects of longer drying time (2 weeks) were tested by placing the samples under a high humidity atmosphere. Investigation of the supercrystal after two weeks of drying revealed a similar internal organization to those obtained after 12 h, indicating that thermodynamic equilibrium was attained at this drying rate. Interestingly, the supercrystal morphology appears to be reminiscent of the shape of the meniscus formed at the triple contact line between the air, the solution and the substrate, as previously reported.^25^ As a consequence, in the latter stage of the drying process the meniscus applies pressure that can deform the supercrystal and affect the local GNR organization. Two parameters were identified to be crucial to obtain a supercrystal with homogeneous organization, namely the cavity volume and the initial particle concentration. We thus conducted experiments on supercrystals obtained from PDMS cavities of smaller lateral dimensions and with varying GNR concentrations, and identified that a large cavity lateral dimension (12 μm) and a high initial GNR concentration ([Au^0^] = 375 mM) were the optimal conditions for our system. On decreasing the width of the square shaped template cavities from 12 to 6 or 2 μm (Fig. 3, S6 and S7†), a reduced long-range organization of the nanorods was observed, suggesting that the increased degree of confinement in cavities with smaller volume hinders the ability of GNR to arrange into organized assemblies. More specifically, GNR organization in supercrystals obtained from 6 μm cavities showed lower directional order (Fig. S6†), whereas GNRs were found to segregate into small supercrystal domains when 2 μm cavities were used, as a result of the predominance of surface cavity effects (Fig. S7†).

**Fig. 4:**
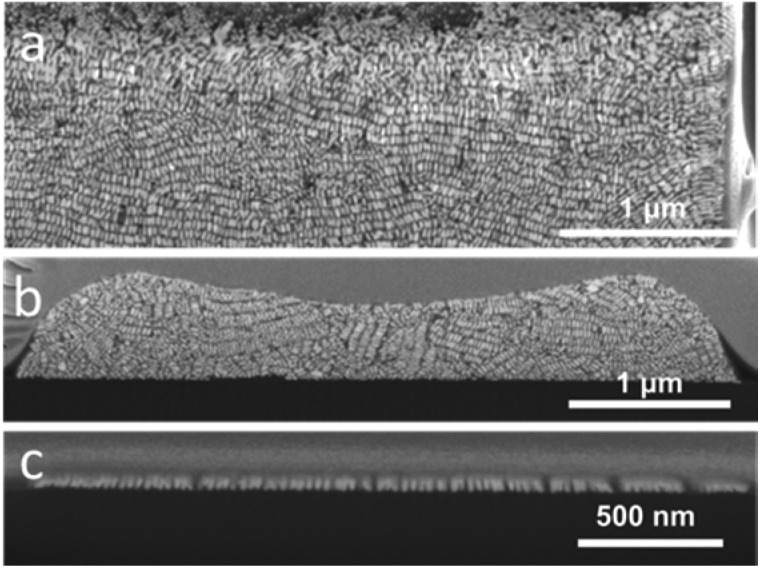
Effect of the gold nanorod concentration on the internal organization. SEM images of internal sections of GNR supercrystals after FIB cutting. GNR concentrations were 375 mM (a), 37 mM (b) and 8.3 mM (c).

Supercrystals obtained from 12 μm wide cavities were thus preferred for this study due to increased long-range organization. For a fixed template cavity size, the initial GNR concentration significantly affects the height of the supercrystal and the extent of internal GNR organization. In Fig. 4, cross-sectional views are shown of typical supercrystals formed with varying GNR concentrations. Different supercrystal heights were thus obtained by changing the GNR concentration: 4 μm, 2 μm, 500 nm and 60 nm (the latter corresponding to a single monolayer of GNR). In supercrystals with 4 μm and 2 μm height, most nanorods were found to align perpendicular to the substrate base, with at least one layer of GNRs perpendicularly oriented with respect to each of the external facets (Fig. 3 and 4a). Supercrystals with a height below 1 μm show no preferential GNR orientation but small domains of parallel rods are observed (Fig. 4b). GNR monolayers standing perpendicular to the substrate were obtained by lowering the GNR concentration (Fig. 4c). As the concentration of GNR was decreased, the larger amount of liquid within the same volume cavity led to perturbations in the long-range order of the GNR domains, possibly resulting from circulating flows in the liquid. In the GNR monolayer, the observed smectic organization, achieved even at low gold concentration, is likely the result of a reduced influence of the walls of the template cavity on the orientation of the nanorods.

In addition to examining the effect of the edge length of square cavities and the GNR concentration on the overall organization, supercrystals obtained from PDMS templates with various cavity shapes were also characterized. For all supercrystal shapes, perpendicular orientation of the rods with respect to the substrate and local organization at the interface were observed, suggesting that interfacial effects are independent of the morphology of the template cavity. Additional SEM images of GNR supercrystal cross-sections are provided in the ESI (Fig. S8–S10†).

### Reinforcement and impregnation of GNR supercrystals with silica

Prolonged immersion (*e.g.* 2 days) of a GNR supercrystal patterned substrate in water was found to lead to partial or even complete detachment of the supercrystals from the surface, leaving a characteristic footprint behind (Fig. S11†). As this may significantly hinder practical applications of such substrates, we aimed at reinforcing the stability of the plasmonic substrates in water by coating them with a protective mesoporous silica film, which would however maintain accessibility of the inner volume from the solution.^42^ Most reports on the synthesis of mesoporous silica films employ spin-coating or dip-coating of a sol–gel solution on the substrate to be covered.^43,44^ In the present case a different approach was followed, in which the silica film was formed by immersing the GNR supercrystal substrates for three days in the silica growth solution. The silica films were formed by hydrolysis of tetraethyl orthosilicate (TEOS) in the presence of cetyl trimethylammonium bromide (CTAB) micelles in a 3 : 1 water : ethanol solution at a basic pH between 9 and 10 (adjusted by the addition of NH_4_OH) (Fig. 1b). The procedure to grow the mesoporous silica film was adapted from a recent report in which the pores were templated by CTAB micelles and oriented perpendicular to a glass substrate.^45^

Optimization of the procedure was first conducted by varying the incubation time, *i.e.* the substrates were removed from the growth solution after one, three and six days of incubation time (Fig. S12†). The silica film thickness on top of the supercrystals increased from 85 to 120 and 150 nm as the growth time was increased. However, after six days of incubation, detachment of a newly formed silica film beneath the supercrystals was noted, indicating an upper limit to the silica growth time for optimum coating (Fig. S13†). The size of the GNR-silica supercrystals was found to increase as compared to that of the starting GNR supercrystals, evidencing the growth of the silica film. GNR supercrystals with a mean width of 7.2 μm expanded to 8.1 μm after 3 days of silica growth, while GNR supercrystals with a mean height of 1.4 μm expanded to 2.0 μm. In the optimized procedure, the supercrystals were immersed for three days in the sol–gel solution so as to achieve a compromise between efficient silica loading and preventing uncontrolled growth (Fig. S14 and S15†). After silica growth, a heat treatment at 130 °C was applied for two hours to improve the stability of the silica in aqueous media. Although in other procedures silica is typically calcined at around 550 °C to consolidate its structure, such high temperatures lead to reshaping and mutual fusion of gold nanorods, thereby altering the plasmonic properties. Optimization showed that 130 °C was the maximum temperature to be used to avoid significant nanorod reshaping (Fig. S16†).

**Fig. 5:**
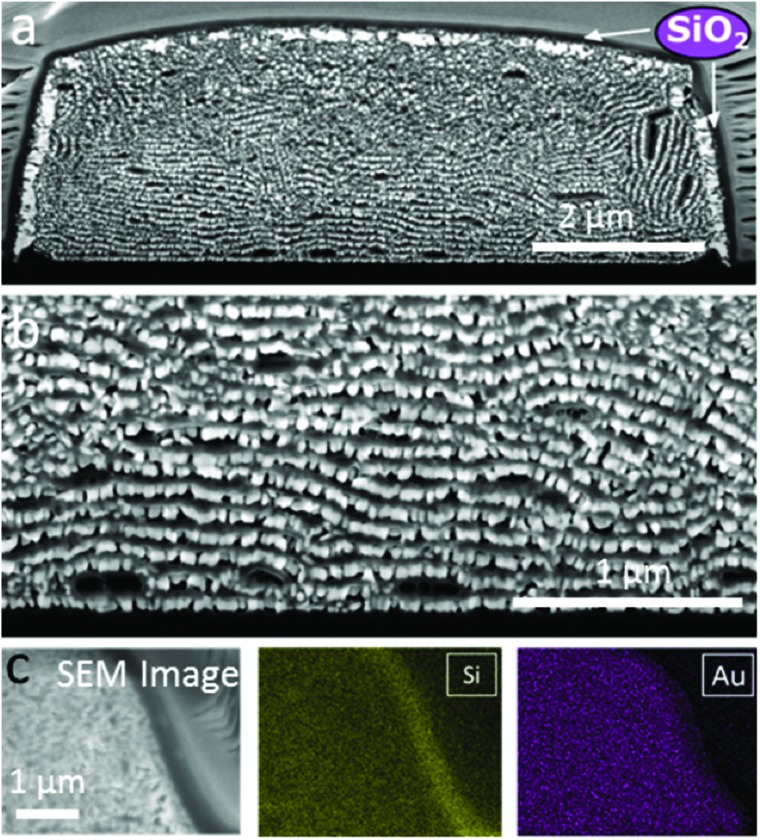
Characterization of GNR supercrystals coated with a mesoporous silica film. (a and b) SEM images of a supercrystal cross section (after the FIB process) at different magnifications. (c) EDX mapping of silicon (Si) and gold (Au) at the edge of a GNR-silica supercrystal.

This sol–gel process resulted in the formation of a silica film on top of the supercrystals but also in the intercalation of silica layers between GNR lamellae, as shown in Fig. 5. FIB-SEM characterization in combination with EDX elemental mapping clearly showed the presence of a uniform silica layer surrounding the supercrystal periphery but also an increased separation between consecutive GNR monolayers (Fig. 5). The silica film uniformly covered the entire supercrystal, as evidenced by 3D volume reconstruction by Slice & View™ (Video 3 and Fig. S17†). Furthermore, image analysis demonstrated that the initial rod organization was unaffected by silica growth as the average orientation of the GNR embedded in silica was 100° ± 20° with respect to the glass substrate (Fig. S18†).

**Fig. 6:**
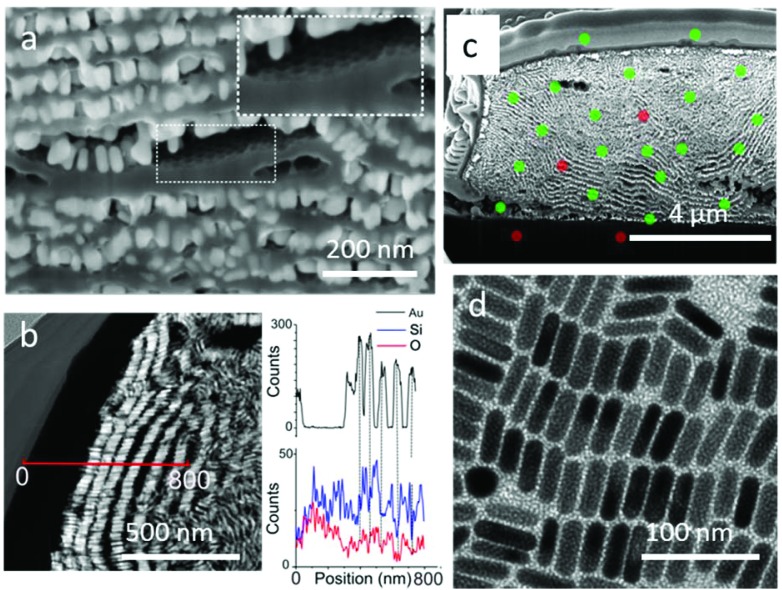
Evidence of the infiltration of porous silica between gold nanorod monolayers. (a) SEM image at high magnification. The inset shows a honeycomb lattice formed in a silica layer as a result of the imprint of the hexagonal GNR lattice. (b) HAADF STEM image of a GNR-silica supercrystal section. The red line indicates the position of the EDX line scan corresponding to the graphs representing the intensity of Au (top), Si and O (bottom) EDX signals as a function of the position. (c) SEM image of a gold–silica supercrystal section incubated with a 1.5% uranyl acetate solution overnight and subsequently cut by FIB. Green spots indicate detection of uranium in EDX whereas red spots indicate that the signal was too low to be detected *via* EDX. (d) TEM images of gold nanorods deposited onto a carbon coated TEM grid by the PDMS driven process and then covered by a mesoporous silica film.

Characterization by high magnification SEM clearly revealed the growth of a film between GNR monolayers with a thickness of around 20 nm (Fig. 6a, Video 4†). Thin lamellae from the supercrystals were extracted for STEM observation with a high angle annular dark field (HAADF) detector (Fig. S19†). HAADF-STEM analysis (Fig. 6b) clearly revealed alternating layers of gold nanorods (bright areas) and lighter elements (dark areas). Complementary EDX line scan analysis confirmed the presence of gold in the bright spots, as well as silica infiltration between GNR layers (Fig. 6b). In some places, the separation of a GNR layer from the silica film left the characteristic imprint of a honeycomb-like pattern, suggesting that silica was growing not only between the GNR monolayers, but also inside (inset, Fig. 6a).

We next aimed to characterize the porosity of the supercrystals. The silica pores could be observed by TEM on the periphery of a GNR supercrystal embedded with silica, directly grown on a microscopy grid (Fig. 6d). The mesochannels were oriented perpendicular to the substrate and their average diameter was measured by electron diffraction to be 2.5 nm. Additionally, some of the structure of the mesochannels was visible through SEM characterization within the supercrystals (Fig. S20†). A thin FIB section of a GNR-silica supercrystal was investigated in TEM but the use of a highly energetic focused ion beam during sample preparation resulted in the collapse of the pores. Therefore, while conclusions cannot be made about the orientation of the silica pores inside the supercrystals, we expect that they are oriented perpendicular to the surface of the GNRs, as observed in Fig. 6d. The porosity of the supercrystal was additionally demonstrated by two experiments aiming at either infiltrating the supercrystal with uranyl acetate or dissolving the gold nanorods by strongly oxidizing aqua regia. The supercrystals were first incubated overnight in a uranyl acetate solution (1.5% in ethanol) and the cross section was investigated by EDX after FIB treatment. The presence of uranium, both at the edge of the supercrystals and inside the structure, was confirmed, demonstrating that uranyl acetate did indeed diffuse through the supercrystal (Fig. 6c and S21†). Note that uranium was not detected below the supercrystals, confirming that the uranium was not redeposited during the FIB process. The experiment with aqua regia resulted in complete GNR dissolution after a short immersion time (5 min), and the formation of hollow micron-sized silica architectures was obtained (Fig. S22†). These experiments confirmed the presence of mesochannels in the silica coating and accessibility of the gold nanorods deep in the composite material, which is significant for applications, as the diffusion of small molecules through the pores to the GNR surface is crucial for sensing applications.

**Fig. 7:**
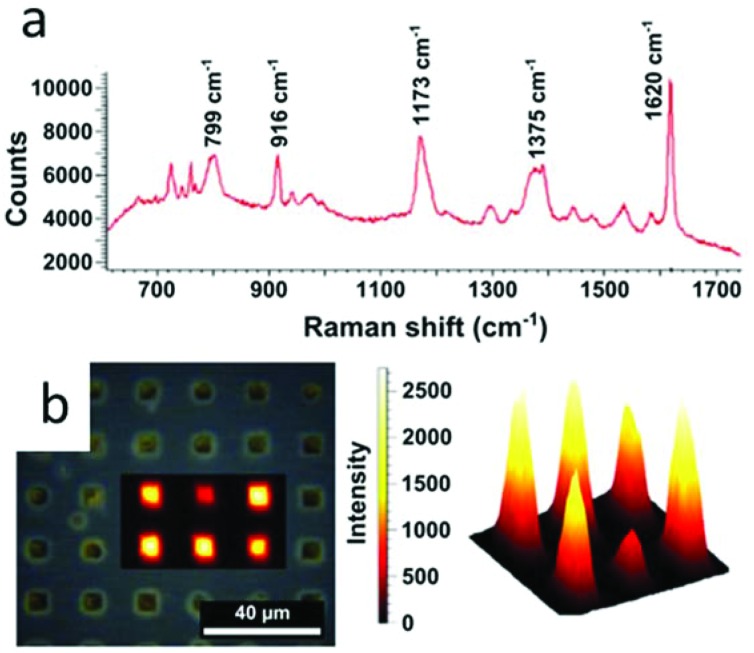
SERRS detection of Crystal Violet on an array of GNR supercrystals coated with mesoporous silica. (a) Typical SERRS spectra of CV measured on the supercrystals. (b) SERRS signal obtained by mapping the SERRS intensity of the crystal violet vibrational peak over 1618–1632 cm^–1^, as observed in a Raman microscope and reconstructed in three dimensions with intensity given as height.

In this context, we examined the sensing properties of GNR-silica supercrystals by means of surface enhanced Raman scattering (SERS). A standard organic pigment, Crystal Violet (CV), was used to demonstrate the efficiency of the composite architectures for SERS sensing. Measurements were performed at an excitation wavelength of 633 nm, which matches the absorption band of CV, resulting in surface enhanced resonant Raman scattering (SERRS). Mapping of the SERRS signal of CV over the supercrystals as well as a typical SERRS spectrum are displayed in Fig. 7. The integrated signal measured across the width of the benzene C–C stretching peak at 1620 cm^–1^ was used to map the presence of CV in the supercrystals (Fig. 7b).^46^ Additional studies using supercrystals formed using templates with other sizes and shapes such as circles, squares, and teardrops were also carried out, and the obtained SERRS maps are provided in the ESI (Fig. S23†). Considering that the pore diameter was estimated to be 2.5 nm, we assume that analytes can diffuse through the silica pores and approach the GNRs inside the supercrystal, where they can experience a large electric field enhancement. We previously reported that the SERRS enhancement factor by a similar GNR assembly without silica was 3.1 × 10^5^ at 633 nm.^25^ In this study, the enhancement factor of the silica-coated supercrystals was calculated to be 7 times that of supercrystals without the silica coating. The superior Raman enhancing capability of the GNR-silica composite supercrystal suggests better accessibility to the internal hotspots through the silica pores. Overall, the strong SERS-enhanced Raman signal observed for CV shows that these substrates are good candidates for use in sensing applications involving small molecule analytes.

## Conclusions

In this work, micropatterned GNR supercrystals were coated and embedded with mesoporous silica to form a composite material with a remarkable SERS enhancement. Gold nanorod organization in the supercrystals was investigated, and long-range smectic ordering was observed in micron sized supercrystals, with long-range order increasing with cavity size and initial concentration of nanorods in solution. Mesoporous silica growth resulted in a uniform coating of silica both inside and outside the supercrystal. Remarkably, silica deposition inside the supercrystal led to a composite material containing alternating layers of silica and gold nanorods organized into lamellae. The mesoporous silica film was shown to allow the diffusion of small ions and organic molecules through the interior of the supercrystal, as demonstrated by uranyl acetate staining and SERS measurements. The composite materials containing organized gold nanorods with silica layers were shown to be effective SERS active substrates, with Raman enhancement factors of CV that are 7 times those of non-infiltrated GNR supercrystals.

Overall, the results presented herein reveal a robust composite material that can be patterned into arbitrary shapes, which shows great promise for sensing applications. Studies are underway involving the application of these substrates for sensing small molecule analytes and biomarkers within complex fluids such as biofluids, which normally represent a challenge for highly sensitive SERS detection.

## Experimental section

### Materials

All chemicals were purchased from Sigma Aldrich and used as received unless otherwise indicated. Milli-Q water (resistivity 18.2 MΩ cm at 25 °C) was used in all experiments.

### GNR synthesis

The preparation and functionalization of GNRs were performed as previously described.^25^ Single crystalline gold nanorods (57 ± 5 nm in length, 17 ± 2 nm in width) were synthesized by a seed mediated method involving the prereduction of HAuCl_4_ with salicylic acid, obtaining a purity higher than 95% of the particle population.^47^ The nanorod synthesis was carried out on the liter scale with no loss of quality, which allowed preparing all substrates from the same GNR batch. Nanorods with an absorption maximum at 780 nm were obtained within 4 h after seed addition. GNRs were then washed by centrifugation (7000 rpm, 40 min, 29 °C) and redispersed in 0.05 M CTAB to remove excess reactants. The GNR concentration was determined from the absorbance at 400 nm.^47,48^ For MUDOL grafting, 5 mL of freshly prepared GNR solution was purified by centrifugation (7000 rpm, 40 min, 29 °C) from excess CTAB and dispersed in an equal volume of Milli-Q water to reach a final surfactant concentration of 1 mM. In a typical experiment, 5 mL of 0.2 mM MUDOL solution was then mixed with the purified GNR dispersion, shaken vigorously and left to incubate for 24 h. Starting from 1 L of the GNR-MUDOL colloid, GNRs were concentrated between 8.3 mM and 375 mM by centrifugation (7000 rpm, 40 min, 29 °C). Several centrifugation/redispersion cycles were needed to achieve 375 mM, first in 50 mL Falcon tubes and then in 2 mL Eppendorf tubes. The obtained MUDOL-GNR solution was stable for months.

### Mold fabrication

The microtextured mold for the supercrystals was manufactured in PDMS (Sylgard 184, Dow Corning), following standard soft lithography techniques.^49,50^ The master was composed of pillars having different shapes and sizes on a 4 inch silicon wafer covered with SU-8 resin. PDMS (10 : 1 elastomer to curing agent) was cured at 60 °C for 2 h. In this work, assembly experiments were performed on <100> silicon wafers.

### GNR supercrystal assembly

Patterned substrates were fabricated by drying a GNR dispersion within an array of micron-sized cavities with a fixed height of 4.8 μm and varying lateral dimensions and shapes, as previously reported.^25^ By adjusting the cavity morphology of the template together with the particle concentration, the morphology and height of the supercrystals can be tuned. In all cases, a 2 μL drop of MUDOL-GNR dispersion with the selected concentration was deposited on the micro-textured PDMS template and then covered by the substrate. The solvent was subsequently allowed to evaporate to dryness (within 12 h) and the template removed (Fig. 1a), resulting in the formation of arrays of GNR supercrystals spread over millimeter-sized areas.

### Description of the silica coating procedure

Prior to silica growth, the substrates were cleaned with a combination of oxygen plasma (2 min, 0.4 mbar, 200 W) and UV/ozone (1 h). Oxygen plasma surface treatment was performed in a low pressure plasma system (PICO, Diener Electronic). UV/ozone surface treatment was performed in a UV/Ozone Cleaner (ProCleaner).

For the mesoporous silica film growth, 17.5 mL of a 6 mM CTAB solution in water was mixed with 7.5 mL of ethanol and stirred at 35 °C for ten minutes. 5 μL of NH_3_ (25 vol%) was added to achieve a pH value between 9 and 10. Finally, 20 μL of TEOS was added dropwise while stirring vigorously. 1 mL aliquots of growth solution were immediately poured into Eppendorf centrifuge tubes, where a clean gold substrate had previously been introduced into each of the tubes. The tubes were subsequently closed and sealed with parafilm to avoid evaporation of the synthesis solution and placed in an oven to incubate at 60 °C for three days. The GNR silica substrates were then rinsed with ethanol and immersed in a 0.1 M HCl solution (in ethanol) for 10 min to get rid of excess CTAB. A further rinsing step in ethanol was performed to remove any trace of HCl. Silica microsphere byproducts were observed depending on the strength of the cleaning procedure employed but extensive rinsing with ethanol was enough to efficiently remove organics and silica spheres by-products. The films were then treated at 130 °C for 2 hours to improve silica stability in water without significant reshaping of the nanorods.

### SERS spectroscopy characterization of patterned substrates

SERS measurements were performed on a confocal scanning Raman microscope (micro-Renishaw InVia Reflex system equipped with Peltier charge-coupled device (CCD) detectors) using a 100× objective (N.A. = 0.85) with an excitation wavelength of 633 nm, 1800 lines per mm diffraction grating. *P* ≈ 0.15 mW, and integration time 100 ms. The laser spot size was in the range of one micrometer in diameter. Samples were soaked in a 1 μM solution of Crystal Violet (Sigma-Aldrich) in ethanol for 45 minutes prior to drying and analysis.

### SEM and FIB characterization

SEM images of Au NRs were obtained using a Helios NanoLab 450S (FEI, The Netherlands) at an accelerating voltage of 1–5 kV and a beam current of 100 pA. EDX analysis was performed at an accelerating voltage of 5–15 kV and beam current up to 1 nA. To obtain information about the internal structure of the supercrystals they were cross-sectioned by FIB and the sections were imaged by SEM. A platinum layer was locally deposited on the top of the supercrystal to protect the surface structures during the FIB process. The typical beam current that has been used for cutting was 50–100 pA at an accelerating voltage of 30 kV, and the final polishing current was 7 pA. To create three-dimensional reconstructions, FIB tomography technique Slice & View™ was used. Series of slices were obtained automatically *via* FEI AutoSlice & View™ G2 software (FEI, The Netherlands). The number of slices was about 200, with an approximate slice thickness of 5 nm. The average time required for milling a series was about 7 hours. Further, the resulting series were processed with Avizo software (FEI, The Netherlands), which allows tilt correction and image alignment using cross-correlation. Image filtering was used to remove artifacts from the cutting with the ion beam. As a result, three-dimensional reconstructions of the supercrystals’ internal structure were obtained.
