# Supplementary material for: Hierarchical organization and molecular diffusion in gold nanorod/silica supercrystal nanocomposites
Source: Nanoscale. 2016 Feb 29;8(15):7914–22. doi: 10.1039/c6nr00712k (PMC5317216; doi:10.1039/c6nr00712k)
Supplement: Supplementary file 1 [file NR-008-C6NR00712K-s001.pdf]

Electronic Supplementary Material (ESI) for Nanoscale.
This journal is © The Royal Society of Chemistry 2016

Electronic Supplementary Information

Hierarchical Organization and Molecular Diffusion in Gold Nanorod/Silica Supercrystal Nanocomposites

Cyrille Hamon[1], Marta N. Sanz-Ortiz[1], Evgeny Modin[2,3], Eric H. Hill[1], Leonardo Scarabelli[1], Andrey Chuvilin[3,4], Luis M. Liz-Marzán[1,4,5]

[1] Bionanoplasmonics Laboratory, CIC biomaGUNE, Paseo de Miramón 182, 20009 Donostia – San Sebastian, Spain.

[2] Electron Microscopy and Image Processing Interdisciplinary Laboratory, Far Eastern Federal University, Sukhanova 8, 690000, Vladivostok, Russia.

[3] Electron Microscopy Laboratory, CIC NanoGUNE Consolider, Tolosa Hiribidea, 76, 20019 Donostia – San Sebastian, Spain.

[4] Basque Foundation of Science, IKERBASQUE, 48013 Bilbao, Spain.

[5] Biomedical Research Networking Center in Bioengineering, Biomaterials, and Nanomedicine, CIBER-BBN, Spain.

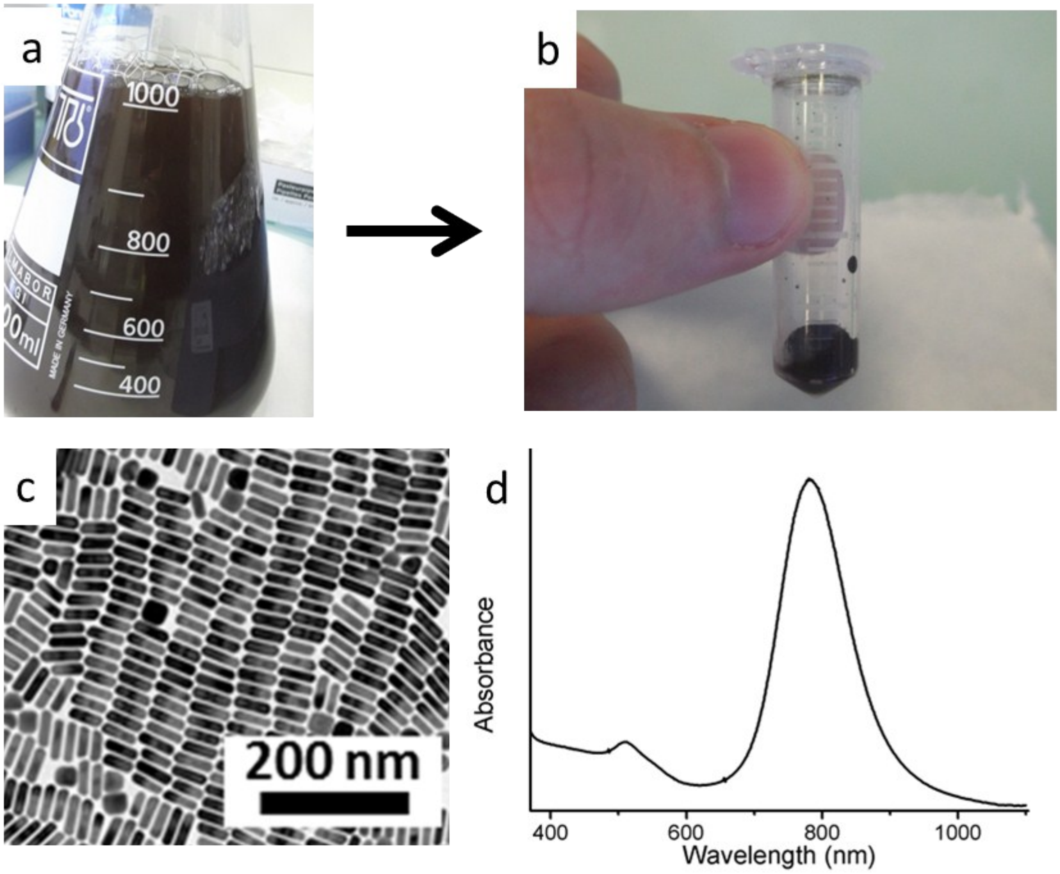

Figure S1. Description of gold nanorods preparation, structure and optical properties. a) Photograph of a 1L erlenmeyer flask in which GNR synthesis was conducted according to previously reported protocols.[1] **b)** After removing excess CTAB, functionalization with MUDOL and concentration of the suspension by centrifugation, a highly concentrated (≈500 μL) colloid was obtained from the 1L batch. **c)** TEM image of the prepared GNRs and **d)** corresponding UV-Vis spectrum.

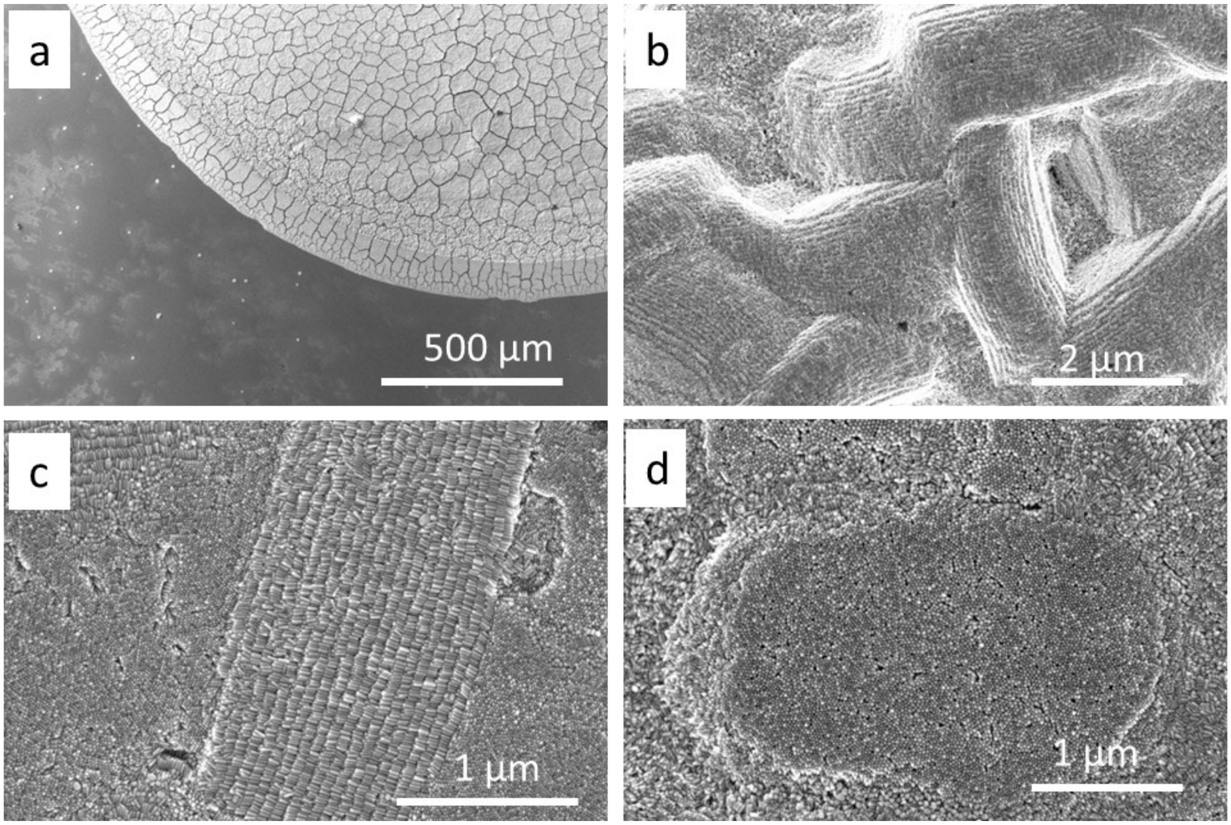

Figure S2. GNR dried without PDMS template. SEM images at various magnifications of GNR assemblies obtained by drop casting. A small volume (2 μL) of MUDOL-GNR solution was drop casted on a silicon wafer. To slow down the evaporation, the system was placed in a homemade setup as previously described.[2] Micron-sized domains of organized GNRs were obtained.

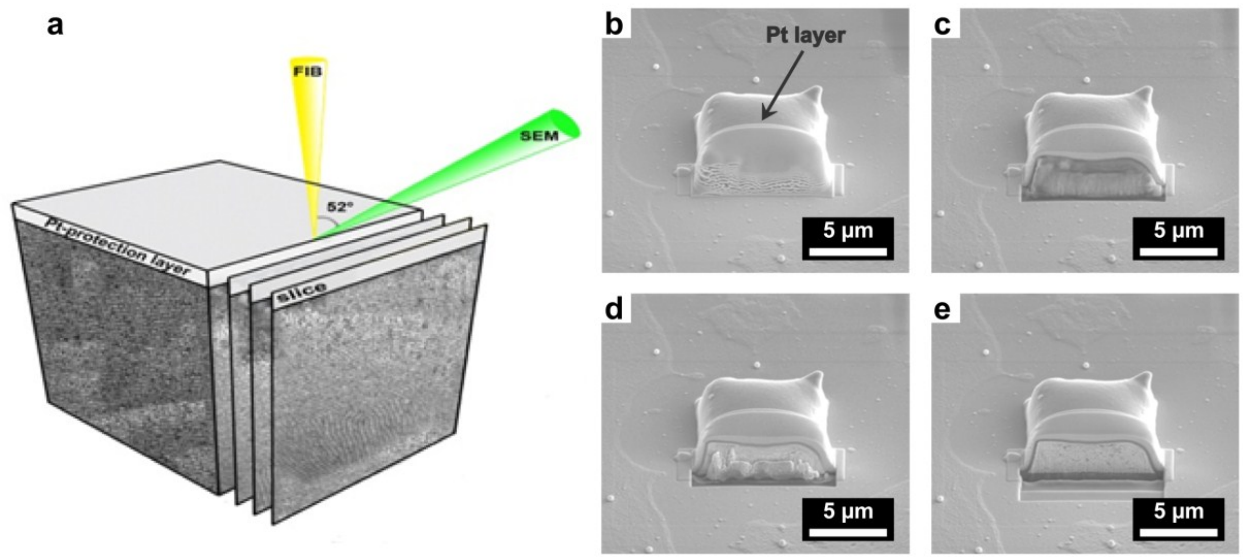

Figure S3. Description of the FIB procedure. **a)** Schematic representation of the Slice&View technique used for the 3D reconstruction of the spatial organization of the gold nanorods within the supercrystals. **b-d)** series of SEM images at different stages of FIB slicing. In **b**, the arrow indicates the protective Pt layer (*i.e.* Methylcyclopentadienyl(trimethyl)Platinum(IV)).

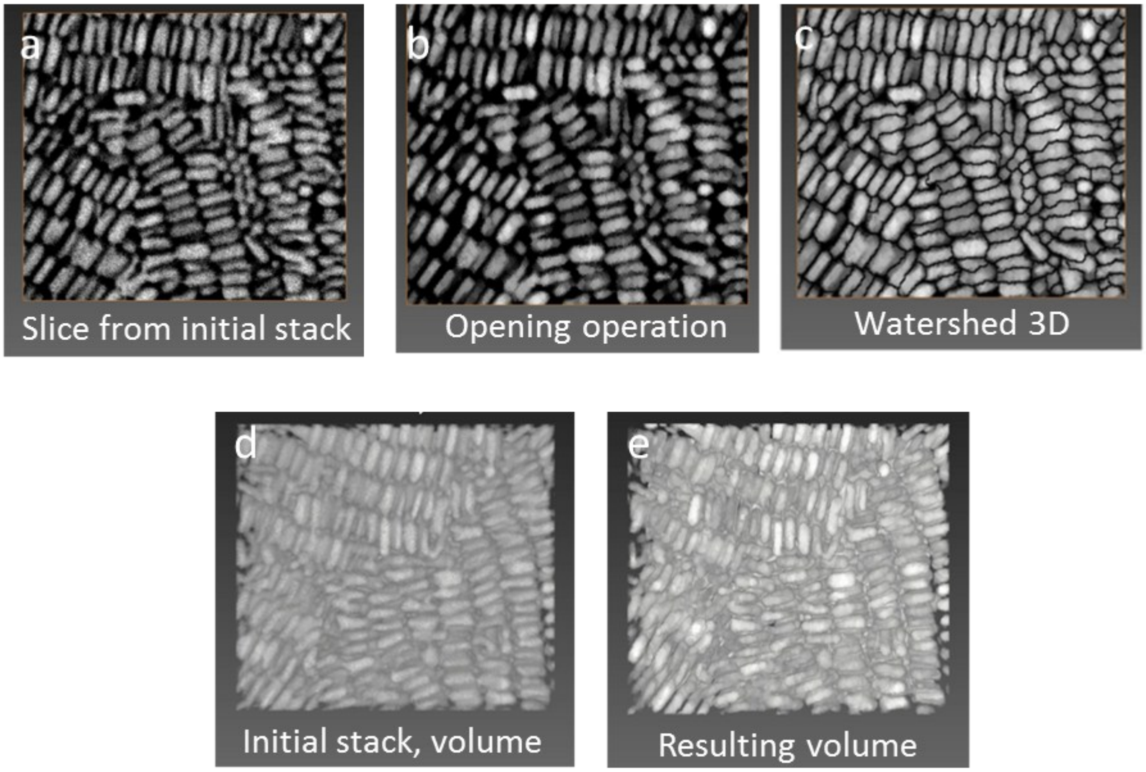

Figure S4. Details about image analysis processing. In order to determine the GNR orientations in volume, image processing was carried out for morphological noise removal: **a)** Non Local Means (noise removal with edge preserving), **b)** 3D-Opening (noise removal and individual rods separation), **c)** 3D-Watershed algorithm for fine rods separation in the volume. **d-e)** Resulting volume reconstructions with well-defined rods, suitable for GNR orientation mapping. The numbers of image stacks were acquired using the SEM-FIB with Auto Slice&View G3 software; volume reconstruction and image processing were held with Avizo 8.1 Fire edition.[3, 4]

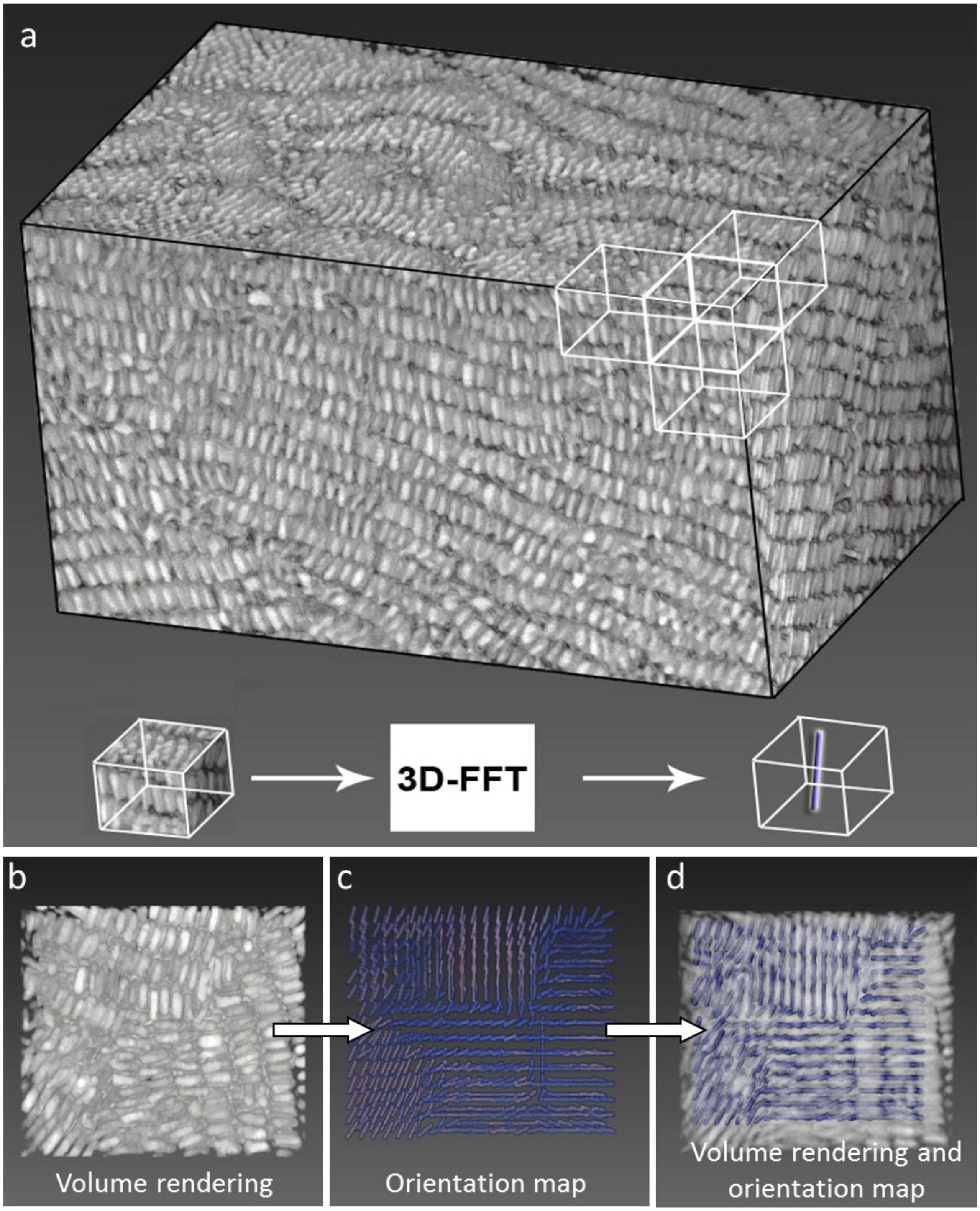

Figure S5. Details about image analysis processing. Supercrystal reconstruction in volume and subsequent image analysis treatment. **a)** To obtain the orientation map, the image is first divided into blocks of defined size of power of 2. The blocks may overlap. **b-c)** The 3-D Fourier transform was then computed on each block to extract the local orientation. As a result we obtain a 3D vector field with well-defined orientation values for each vector. **d)** Overlay of volume rendering of the initial structure and vector field model (orientation map) shows good agreement.

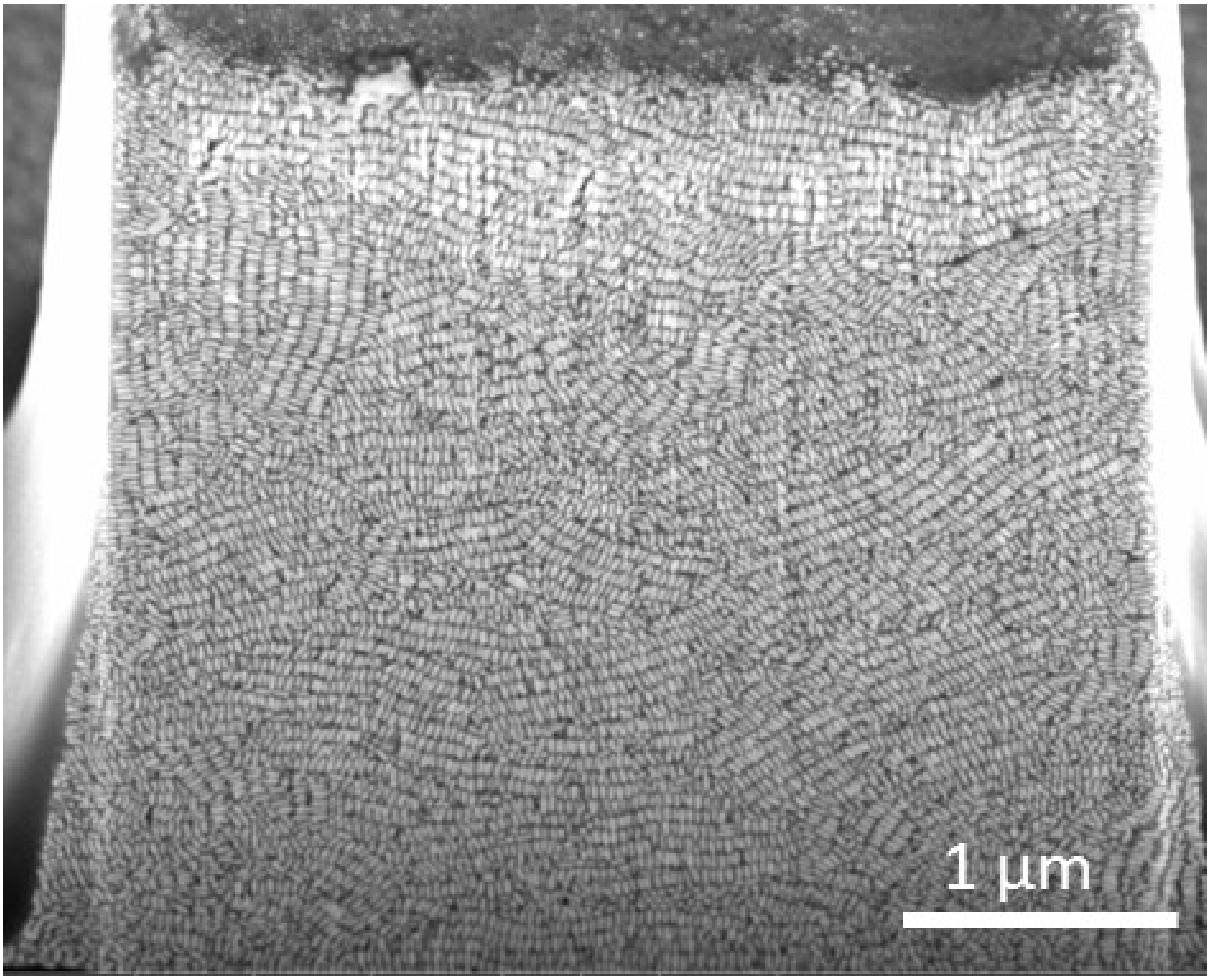

Figure S6. Characterization of GNR supercrystal nanostructure obtained from a cavity of 6 µm and initial Au^0 concentration of 375 mM.

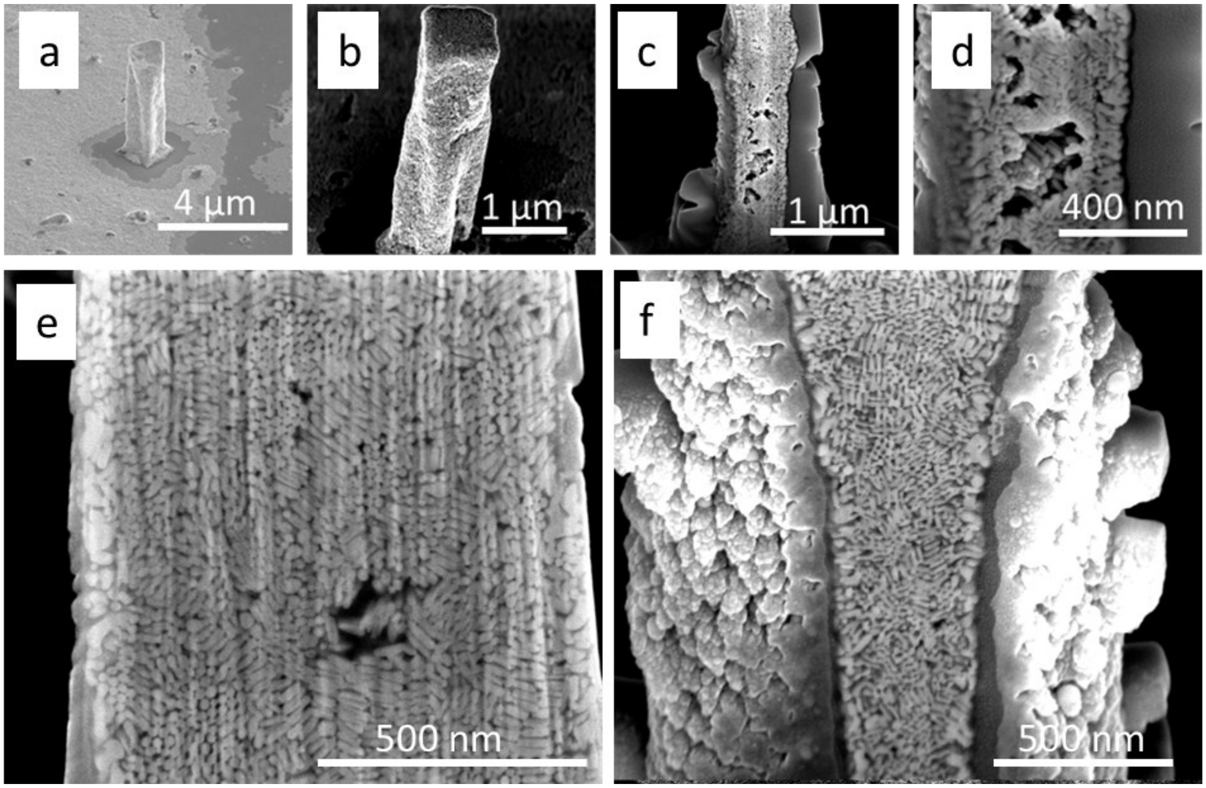

Figure S7. Characterization of GNR supercrystal nanostructure obtained from a cavity of 2 µm and initial Au^0 concentration of 375 mM. SEM images of GNR supercrystal surface **(a-b)** and GNR supercrystal cross-sections **(c-f)**. Short-range GNR organization was observed.

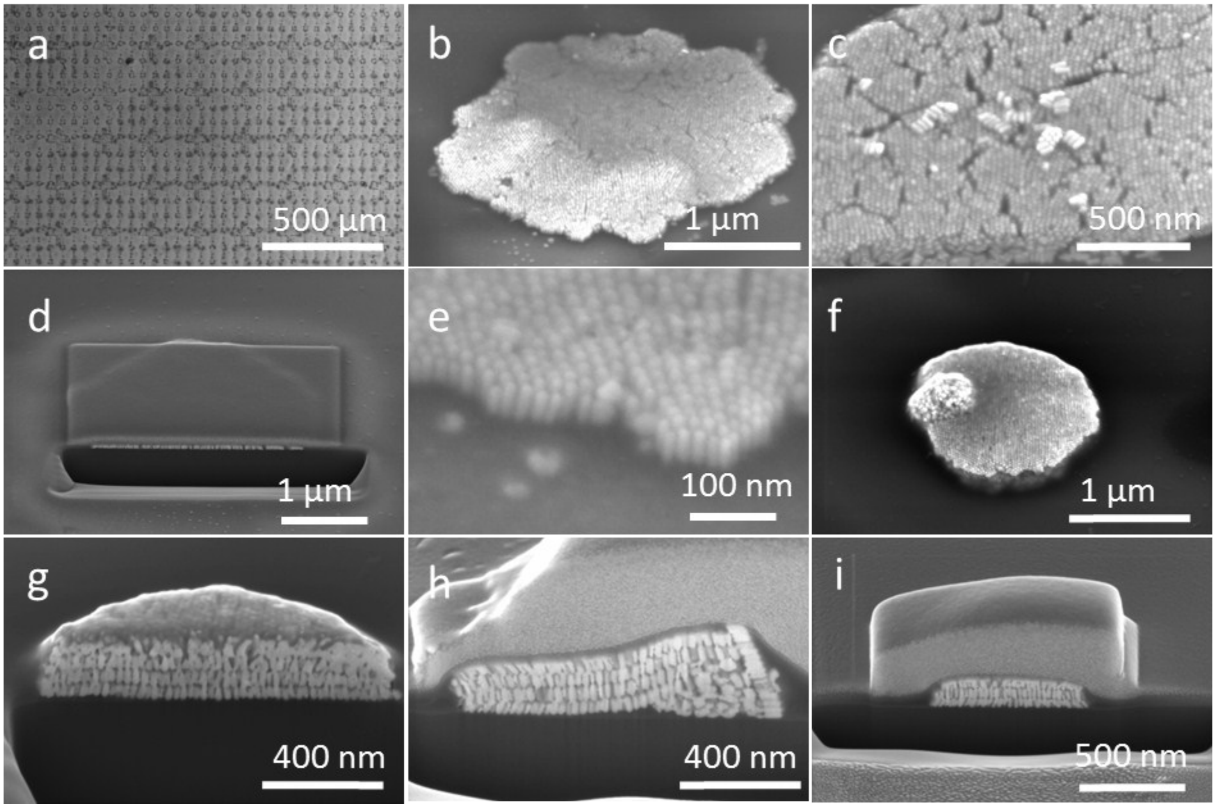

Figure S8. Characterization of GNR supercrystal nanostructure obtained from various cavity shapes and initial concentration in Au^0 of 8.3 mM. SEM images of supercrystals at different magnifications. Supercrystals with few GNR monolayers were observed.

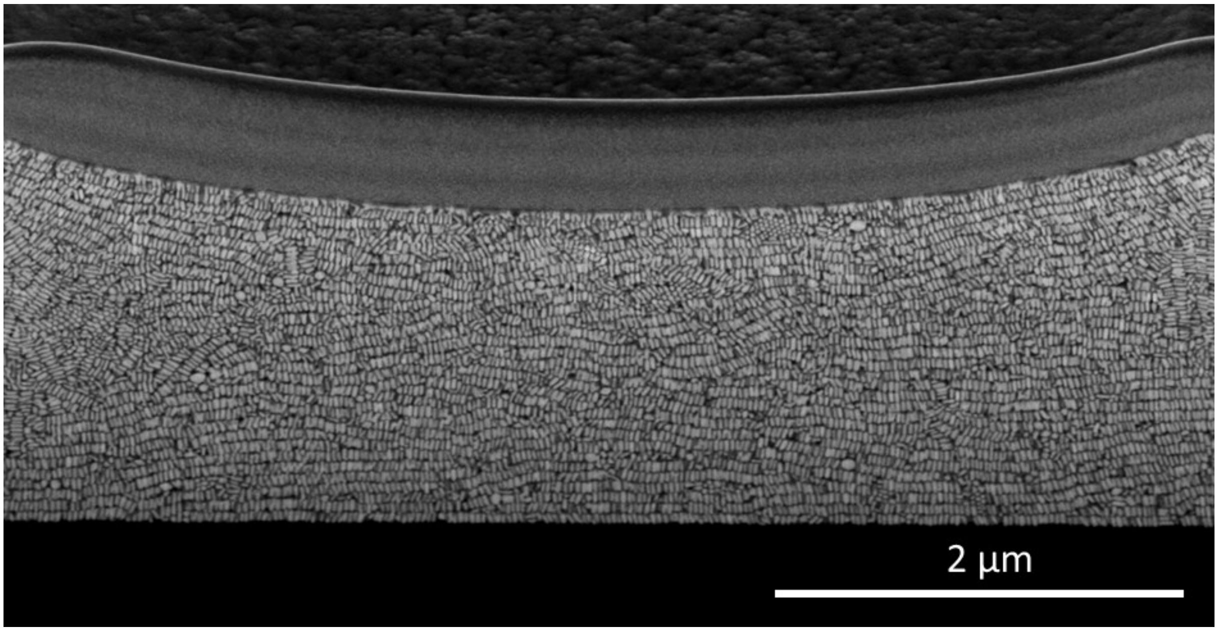

Figure S9. Additional SEM image of the supercrystal displayed in Figure 3 of the manuscript.

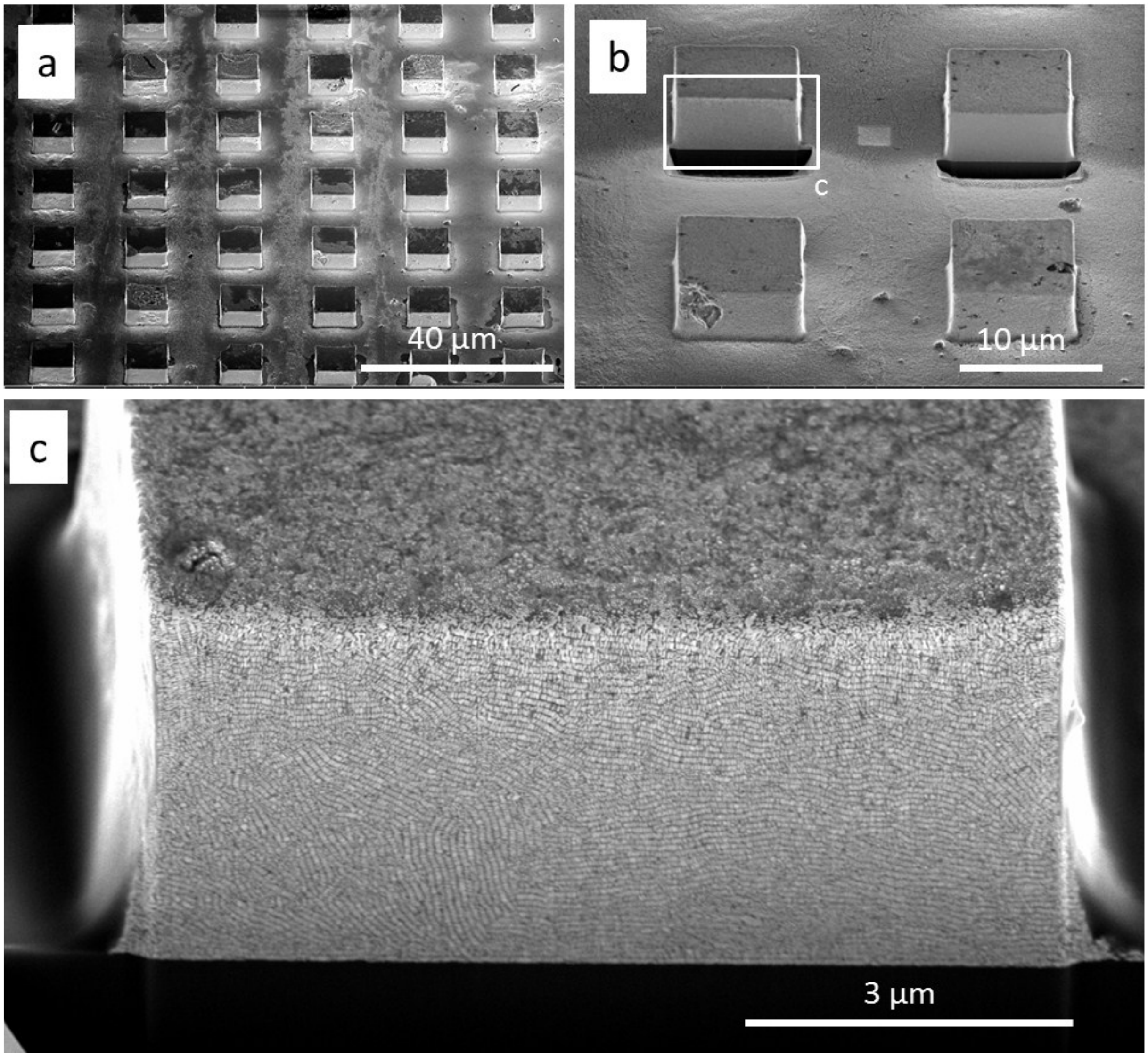

Figure S10. Characterization of GNR supercrystal nanostructure obtained from a 12 µm cavity shape and initial Au^0 concentration of 375 mM. SEM images at different magnifications. Large domains of GNR with perpendicular orientation to the substrate were observed.

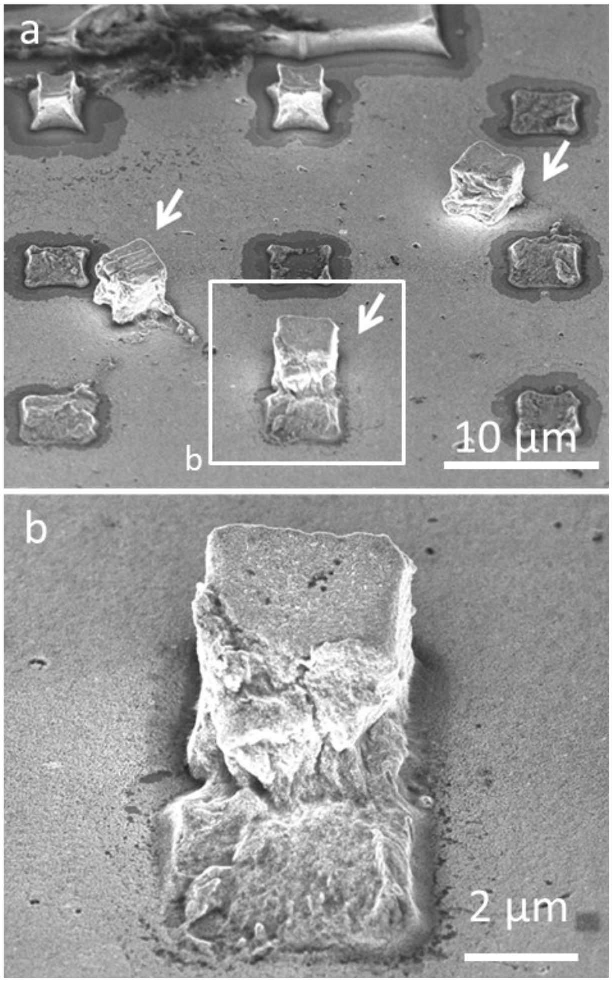

Figure S11. Characterization of the substrates after immersion in water for two days. SEM images at different magnifications. Detachment of the supercrystals from the substrate was observed.

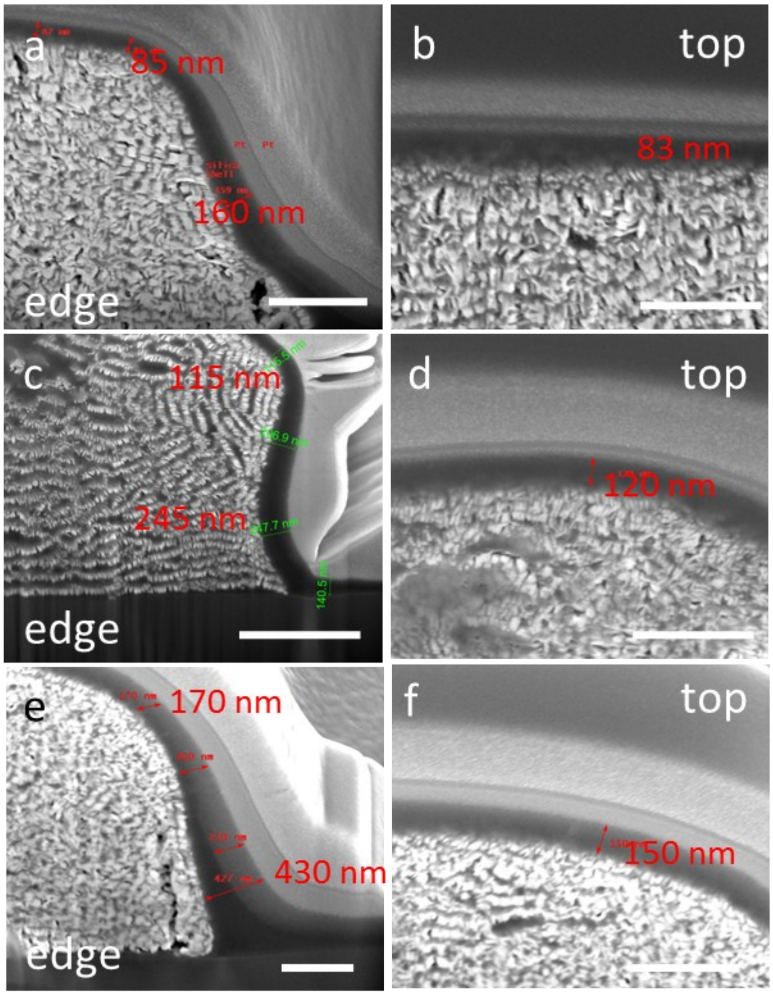

Figure S12. Determination of silica film thickness on top of GNR-silica supercrystals after 1, 3 and 6 days growth time. SEM images obtained from the top and the edge of the supercrystals. **a-b)** GNR-silica supercrystal after 1 day; **c-d)** GNR-silica supercrystal after 3 days; **e-f)** GNR-silica supercrystal after 6 days. The silica film was measured to be thicker on the base of the supercrystal as compared to the top. Corresponding measurements are directly noted on the images.

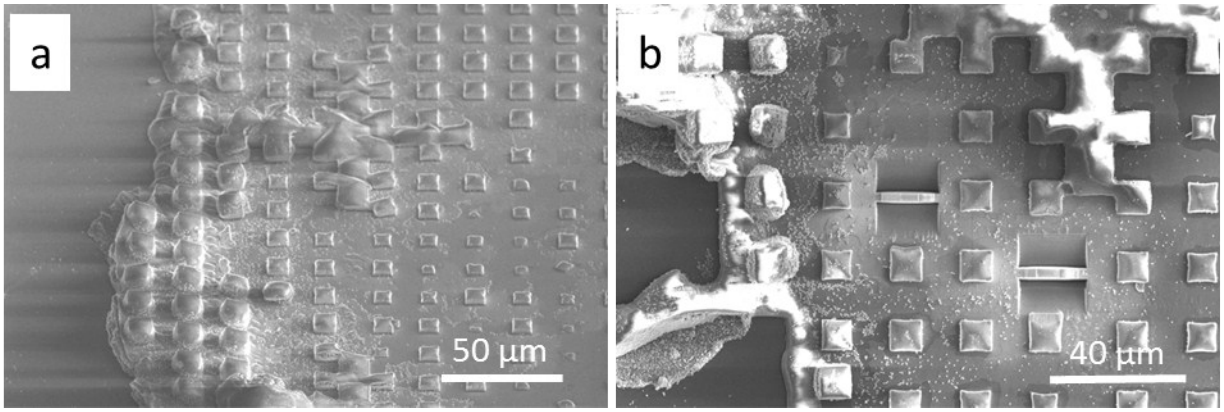

Figure S13. Uncontrolled growth of silica film after 6 days in a silica growth solution. SEM images at different magnifications of GNR-silica supercrystals after 6 days immersed in a silica growth solution. **a)** A thick silica film was noted between the supercrystals and the substrate, as well as detachment of a part of the pattern. **b)** Two lamellas extracted for subsequent TEM observations are visible on the image.

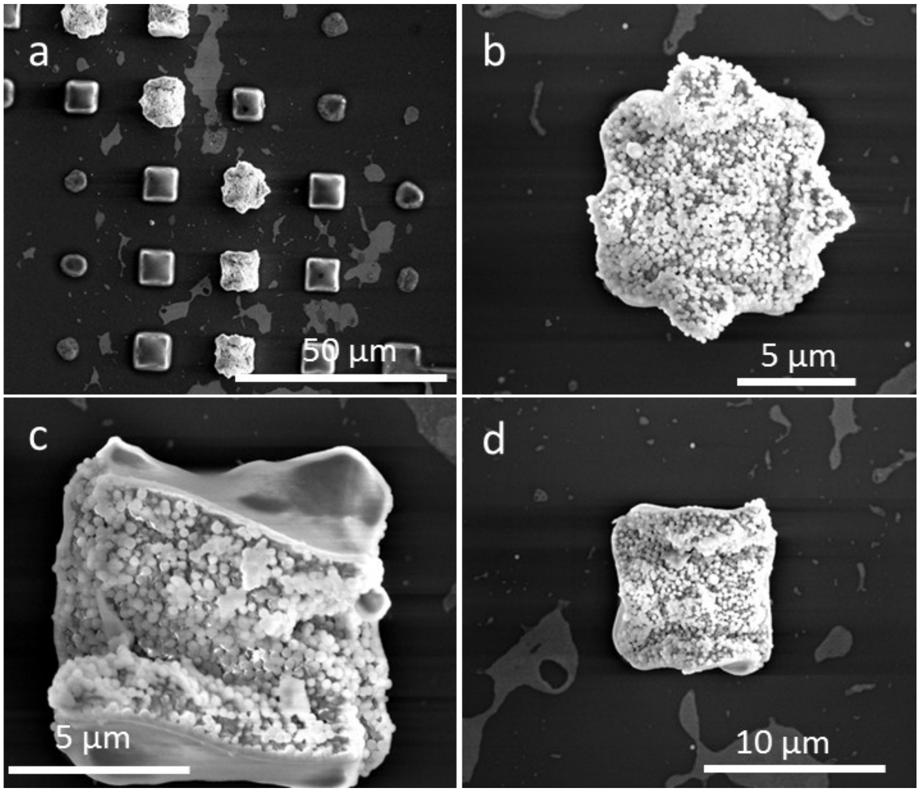

Figure S14. Anomalous silica growth on supercrystals. SEM images of various supercrystals at different magnifications. Top view of GNR-silica supercrystal, showing nucleation of silica microspheres.

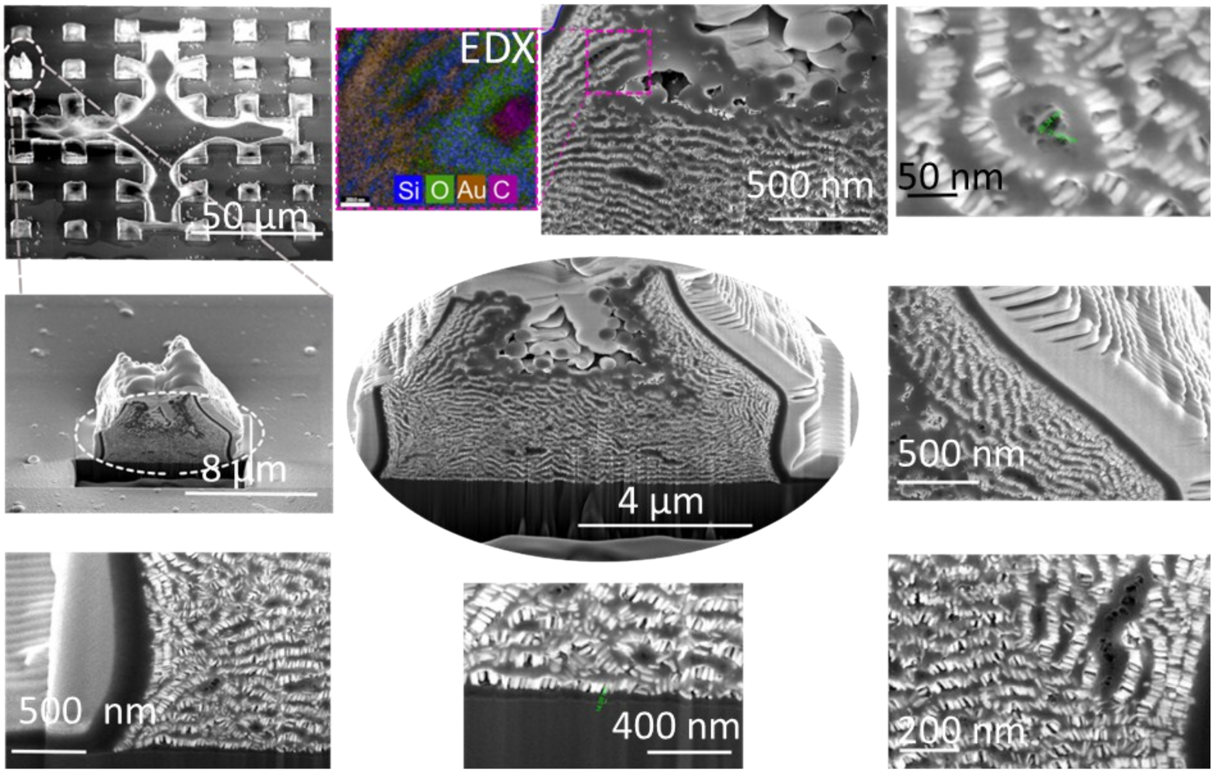

Figure S15. **Characterization of a supercrystal with unusual silica growth.** SEM images of a cross section of a GNR-silica supercrystal showing an anomaly. Silica microspheres were likely to nucleate inside the supercrystal leading to deformation of the top surface.

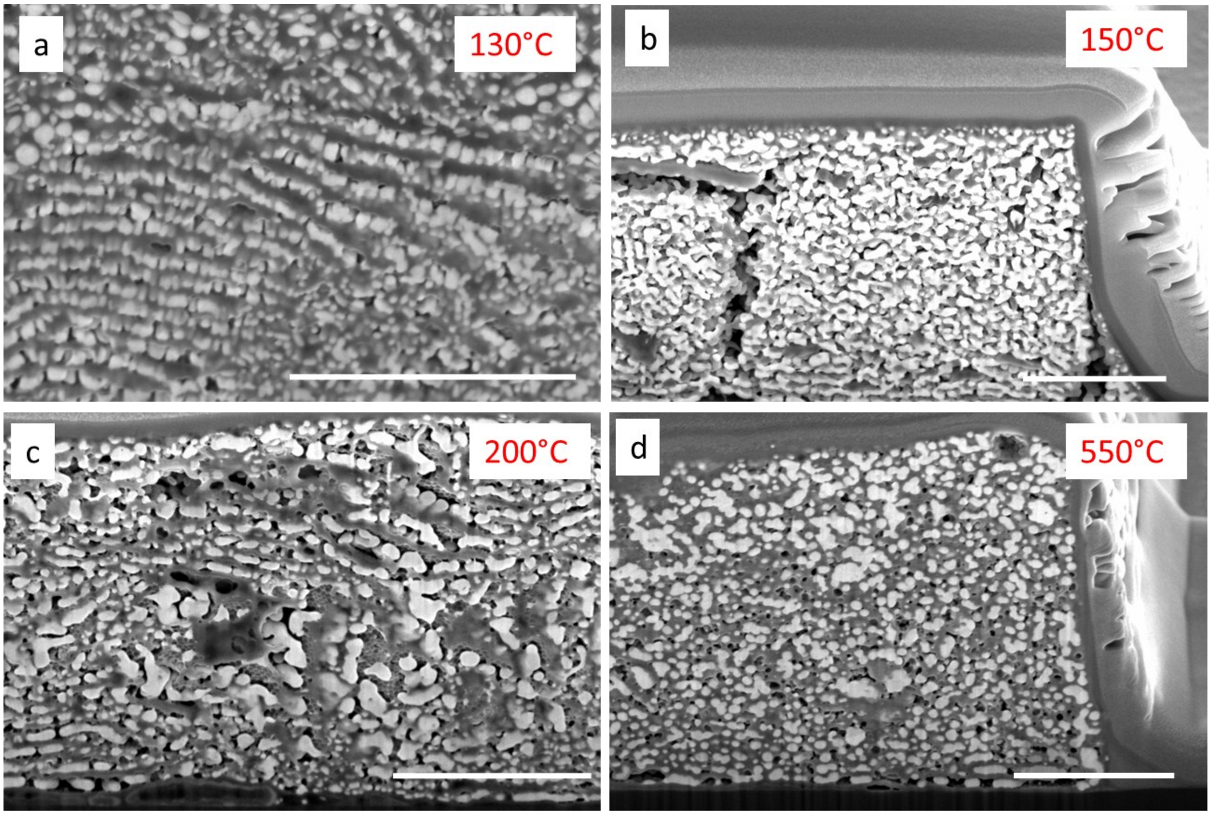

Figure S16. Characterization of supercrystal nanostructure after annealing substrates from 130 °C to 550 °C. a-d) SEM images of a supercrystal annealed at 130 °C **(a)**; 150 °C **(b)**; 200 °C **(c)**; 550 °C **(d)**. Scale bars on all images are 1 µm. Melting of GNR was observed above 150 °C. Temperature increase led to gold reshaping into more isotropic morphology.

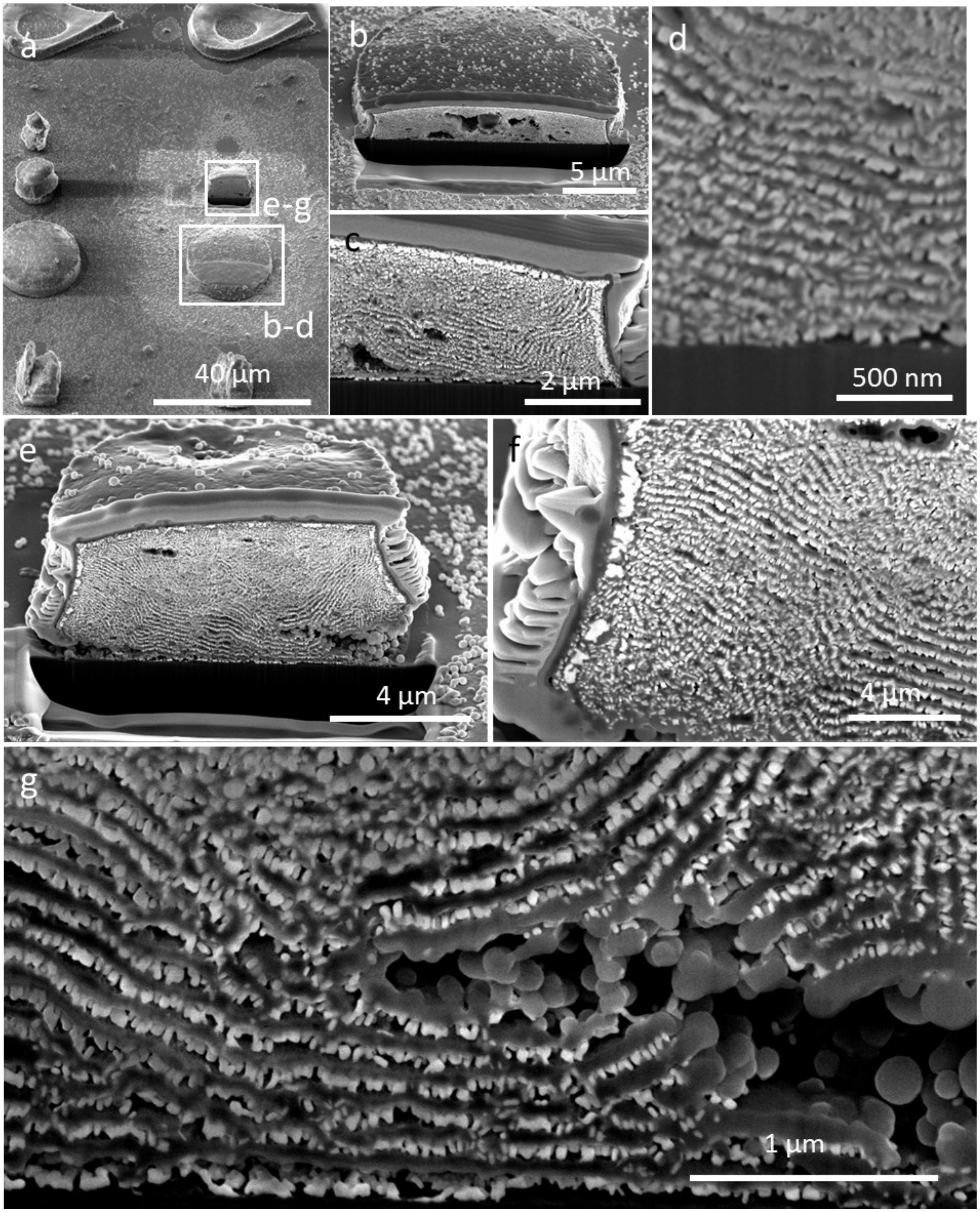

Figure S17. Additional characterization of silica-GNR supercrystals. SEM images of two different GNR-silica supercrystals of 20 μm **(a-d)** and 8 μm **(e-g)** in width.

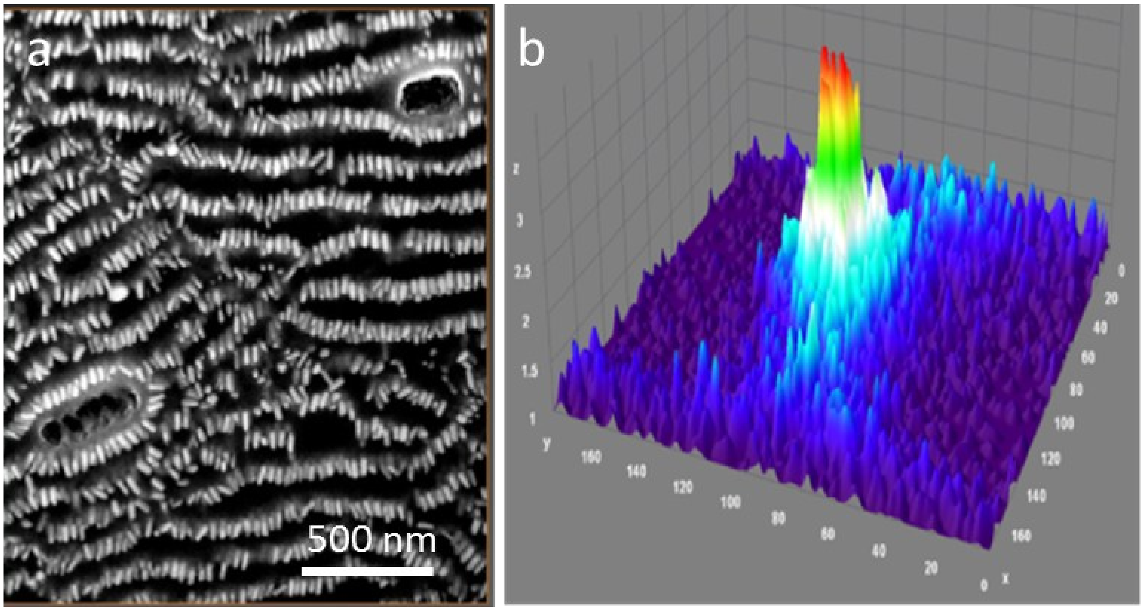

Figure S18. Image of a GNR silica supercrystal section and corresponding 3D orientation scattering plot. a) SEM image and **b)** orientation scattering plot obtained from the analysis of the image in a), revealing the distribution of GNR orientation to be 100º ± 20° with respect to the substrate.

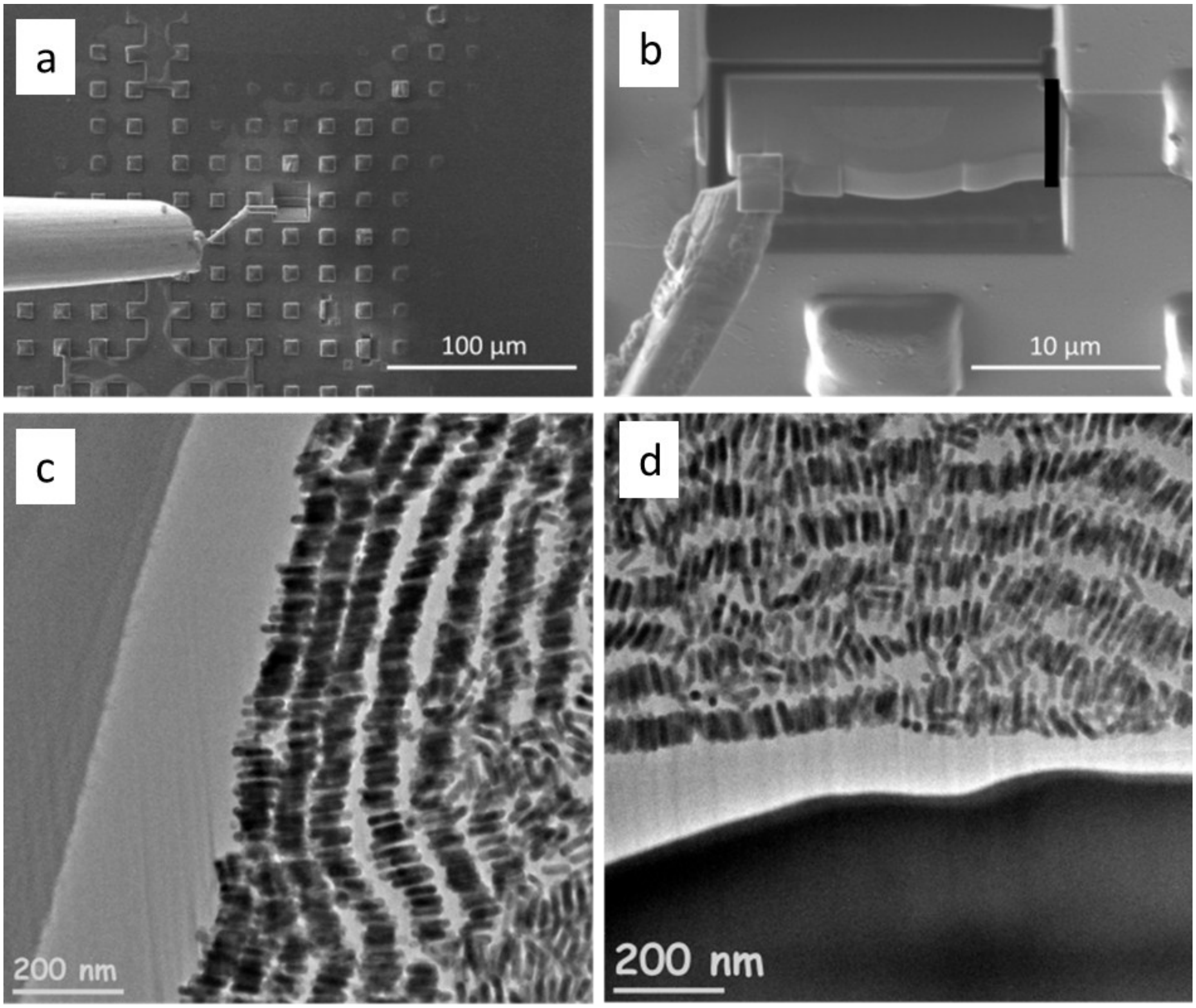

Figure S19. a-b) Extraction of a supercrystal slice with a micromanipulator for TEM observation. **c-d)** TEM images in bright mode of a thin GNR-silica supercrystal cross section.

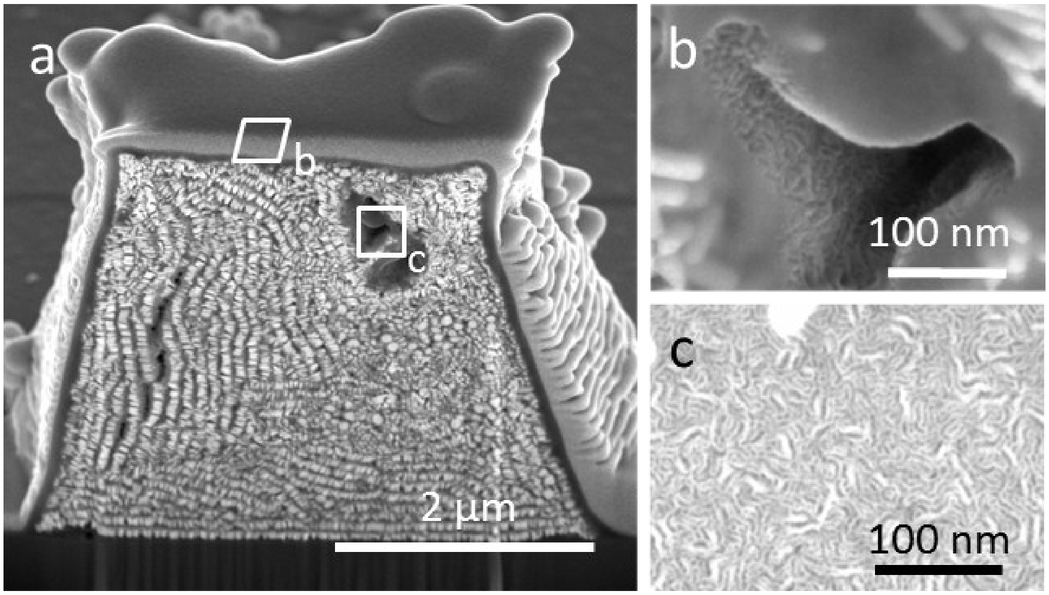

Figure S20. Observation of the mesochannels in a GNR silica supercrystal. SEM images at different magnifications of a GNR silica supercrystal. Channels are visible in **(c)**.

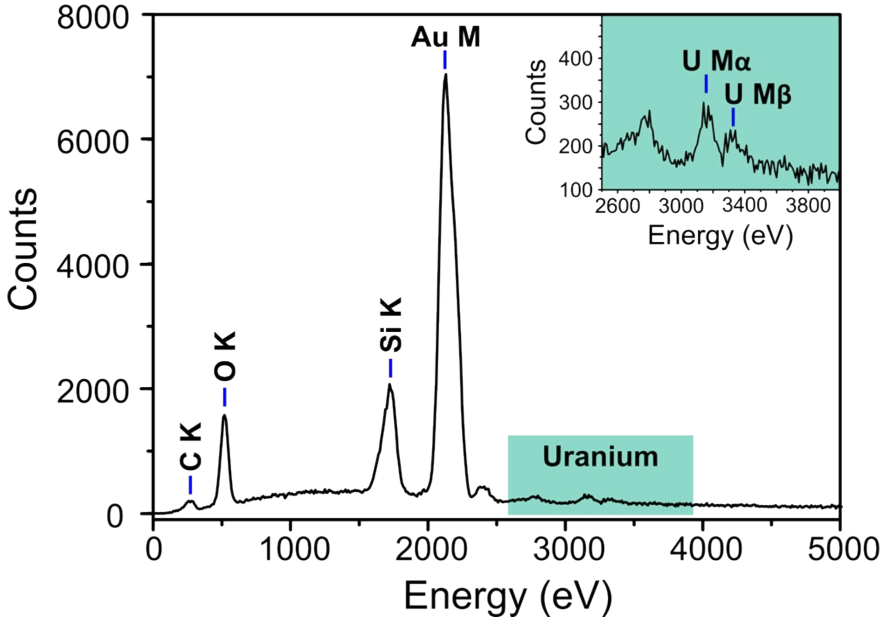

Figure S21. Typical EDX spectrum measured at the green spots in Figure 6c.

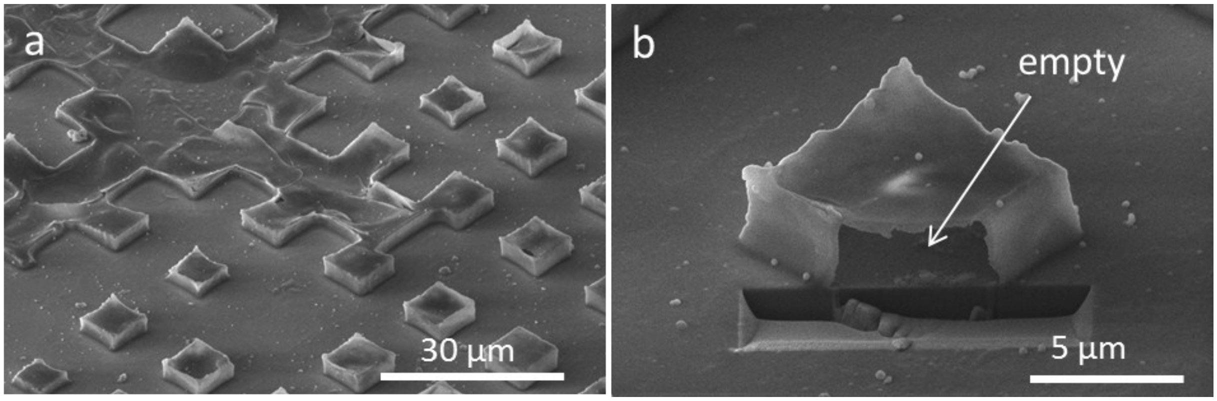

Figure S22. Dissolution of the GNR by *aqua regia*. SEM images of a substrate after treatment with *aqua regia*. The shape of the supercrystals remained unchanged but all the gold was dissolved forming empty silica boxes.

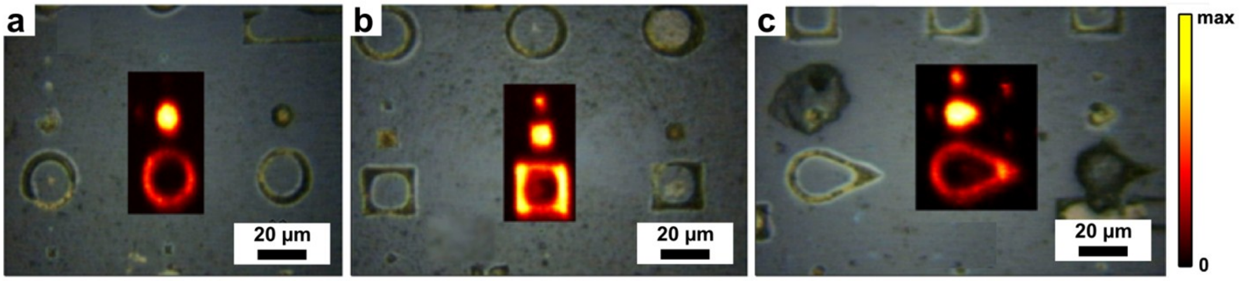

Figure S23. SERS mapping of GNR-silica supercrystals templated into shapes of circles **(a)**, squares **(b)**, and teardrops **(c)**.

References:

1. L. Scarabelli, M. Grzelczak and L. M. Liz-Marzán, *Chem. Mater*. 2013. **25**, 4232-4238.

2. C. Hamon, S. Novikov, L. Scarabelli, L. Basabe-Desmonts and L. M. Liz-Marzán, *ACS Nano*, 2014, **8**, 10694-10703.

3. G. Biener, E. Vrotsos, K. Sugaya and A. Dogariu, *Opt. Express*, 2009, 17, 9724-9732.

4. M. Sivaguru, S. Durgam, R. Ambekar, D. Luedtke, G. Fried, A. Stewart and K. C. Toussaint, *Opt. Express*, 2010, **18**, 24983-24993.
